# Vulnerabilities and reparative strategies during pregnancy, childbirth, and the postpartum period: moving from rhetoric to action

**DOI:** 10.1016/j.eclinm.2023.102264

**Published:** 2023-12-06

**Authors:** Jameela Sheikh, John Allotey, Tania Kew, Halimah Khalil, Hadiza Galadanci, G Justus Hofmeyr, Edgardo Abalos, Joshua P. Vogel, Tina Lavin, João Paulo Souza, Inderjeet Kaur, Uma Ram, Ana Pilar Betran, Meghan A. Bohren, Olufemi T. Oladapo, Shakila Thangaratinam

**Affiliations:** a College of Medical and Dental Sciences, University of Birmingham, Birmingham, United Kingdom; b WHO Collaborating Centre for Global Women’s Health, Institute of Metabolism and Systems Research, University of Birmingham, Birmingham, United Kingdom; c National Institute of Health and Care Research (NIHR) Birmingham Biomedical Research Centre, Birmingham, United Kingdom; d Africa Center of Excellence for Population Health and Policy, College of Health Sciences, Bayero University, Kano, Nigeria; e Department of Obstetrics and Gynaecology, University of Botswana, Gaborone, Botswana; f University of the Witwatersrand and Walter Sisulu University, East London, South Africa; g Centro de Estudios de Estado y Sociedad (CEDES), Buenos Aires, Argentina; h Maternal, Child and Adolescent Health Program, Burnet Institute, Melbourne, Australia; i UNDP/UNFPA/UNICEF/WHO/World Bank Special Programme of Research, Development and Research Training in Human Reproduction (HRP), Department of Sexual and Reproductive Health and Research, World Health Organization, Geneva, Switzerland; j Department of Social Medicine, Ribeirão Preto Medical School, University of São Paulo, Ribeirão Preto, São Paulo, Brazil; k BIREME, Evidence and Intelligence for Action in Health Department, Pan America Health Organization/World Health Organization, São Paulo, Brazil; l Fernandez Hospital Educational & Research Foundation, Hyderabad, India; m Seethapathy Clinic & Hospital, Chennai, India; n Gender and Women’s Health Unit, Nossal Institute for Global Health, School of Population and Global Health, The University of Melbourne, Victoria, Australia; o Birmingham Women’s Hospital, Birmingham Women’s and Children’s NHS Foundation Trust, Birmingham, United Kingdom

**Keywords:** Vulnerability, Pregnant, LMIC

## Abstract

Maternal outcomes throughout pregnancy, childbirth, and the postnatal period are influenced by interlinked and interdependent vulnerabilities. A comprehensive understanding of how various threats and barriers affect maternal and perinatal health is critical to plan, evaluate and improve maternal health programmes. This paper builds on the introductory paper of the Series on the determinants of maternal health by assessing vulnerabilities during pregnancy, childbirth, and the postnatal period. We synthesise and present the concept of vulnerability in pregnancy and childbirth, and map vulnerability attributes and their dynamic influence on maternal outcomes in early and late pregnancy and during childbirth and the postnatal period, with a particular focus on low-income and middle-income countries (LMICs). We summarise existing literature and present the evidence on the effects of various reparative strategies to improve pregnancy and childbirth outcomes. Lastly, we discuss the implications of the identified vulnerability attributes and reparative strategies for the efforts of policymakers, healthcare professionals, and researchers working towards improving outcomes for women and birthing people in LMICs.

**Key messages**

• The term ‘vulnerability’ is ubiquitous in global health, but the meaning is quite diverse, and its application is vague. In maternal health, vulnerabilities have primarily been used solely as markers of susceptibility to potential harm or social determinants during pregnancy, childbirth, and the postpartum period.
• Vulnerability in maternal health is centred around threats, barriers, and reparations, all in constant interactions with each other and determining the woman’s vulnerability trajectory along the pregnancy journey, where vulnerability attributes can appear, disappear, change, or reinforce at any point through the continuum.
• Key threats and barriers accounting for the disproportionally high maternal morbidity and mortality in LMICs include adolescent pregnancy, primigravidity, nutritional deficiencies, low education levels and migrant and refugee status. Notable reparative strategies include iron and calcium supplementation, community-based educational interventions, financial incentive programmes, and contraception-promoting interventions.
• Policymakers, healthcare professionals, and researchers have an ethical obligation to use the vulnerability concept to improve pregnancy outcomes—using a preventative lens and a life course approach—to rationalise increased resources for maternal health, identify vulnerability and customise care plans in maternity settings, and evaluate strategies to scale up effective interventions.

## Introduction

Health vulnerability is a state where an individual (or a group of individuals) is exposed to physical, psychological, cognitive, and or social risk factors in the context of a lack of adequate support or coping strategies to neutralise potential adverse effects of these risk factors.^1^^,^^2^ For maternal health, this translates to inherent physical and physiological changes that make women’s bodies susceptible to certain health conditions (e.g., anaemia, gestational diabetes), the emotional and psychological challenges associated with pregnancy and transition to motherhood (e.g., anxiety, depression), and limited access to healthcare, education, and economic resources (e.g., among socially marginalised populations). This is in combination with a lack of appropriate strategies to mitigate the risk of adverse outcomes, including severe morbidities and death. The impact of vulnerability during pregnancy and childbirth is particularly relevant to women in low-income and middle-income countries (LMICs), where the burden of maternal and perinatal mortality and morbidity is disproportionately high.^3^ Drivers of vulnerability, however, also disproportionately impact women from socially marginalised groups in any setting, such as those in high-income countries who are Indigenous or from migrant or refugee backgrounds.^4^, ^5^, ^6^

But application of the vulnerability concept to clinical practice and public health is often vague, particularly in maternal health, leading to a wide variety of interpretation (and misinterpretation) of the concept.^2^^,^^7^ This can result in tokenistic characterisations of individuals, groups, or communities who are susceptible to maternal ill-health and disability. Such vague depictions of vulnerability around pregnancy and childbirth mean that pregnant and postpartum women, healthcare professionals, and policymakers do not have the necessary guidance to meaningfully apply the concept to improve maternal and perinatal outcomes.^1^^,^^2^ A person-centred approach to identifying key vulnerability attributes in pregnant women can help design and implement targeted reparative interventions to restore and improve pregnancy and childbirth outcomes, halt perpetuation of vulnerability experienced during pregnancy throughout the life course for the woman, and mitigate its intergenerational impacts.

The multifaceted (and challenging) nature of vulnerability is evident in the interlinkages between factors that adversely affect women, spanning across the continuum of pregnancy through to the postpartum period and beyond. For example, poor outcomes for women and babies can arise from worsening pre-existing conditions like hypertension and anaemia, which can predispose to complications like pre-eclampsia and postpartum haemorrhage, respectively.^8^^,^^9^ Pre-eclampsia contributes significantly to perinatal mortality through its linkages to fetal growth restriction and preterm birth and to maternal mortality—not just from eclampsia but also by predisposing the woman to caesarean section with its increased risk of postpartum haemorrhage from pre-eclampsia-induced coagulopathies.^10^, ^11^, ^12^, ^13^ Mental health issues like depression during pregnancy can increase the risk of complications such as preterm birth, low birthweight, and developmental delays in the offspring.^14^ Small vulnerable newborns including preterm, small-for-gestational-age, and low birthweight babies are in turn, at higher risk for lifetime health and developmental problems (e.g., hypertension, coronary disease and stroke in adulthood).^10^ Therefore, efforts to mitigate threats posed by a single condition without considering the ramifications throughout the entire pregnancy journey and beyond are inadequate to ensure the health and wellbeing of the woman and her baby and represent missed opportunities to use a life course approach to break the vicious cycle of vulnerability.

In this second paper of the Series, we systematically review the literature and map the concept of vulnerability and its dynamic attributes during pregnancy, childbirth and the postpartum period using a vulnerability framework. We provide an evidence-based overview of the impact of various vulnerability attributes on maternal health during early and late pregnancy, childbirth, and the immediate postpartum period, specifically for LMICs. We apply the vulnerability framework to specific conditions and complications associated with adverse pregnancy outcomes to highlight how the interlinked vulnerability attributes impact outcomes for women across various stages of pregnancy, childbirth, and the postpartum period in LMICs. We report on the effects of reparative strategies in addressing the threats and barriers posed by various vulnerability attributes and highlight the implications of vulnerability and reparation for maternal health research, clinical practice, and policy. In this paper, the terms ‘woman’ and ‘women’ are used to reflect all populations with the reproductive capacity for pregnancy and birth, including transgender and gender-diverse people, as well as adolescent girls.

## Vulnerability concepts, attributes and models related to pregnancy, childbirth, and the postpartum period

Using the criteria in Panel 1, we searched for systematic reviews on vulnerability concepts to identify vulnerability attributes related to pregnancy and childbirth outcomes. We identified four systematic reviews that defined vulnerability concepts, attributes, and or conceptual models in the context of pregnancy and childbirth.^2^^,^^7^^,^^15^^,^^16^ Briscoe et al. (26 studies) reported three main categories of vulnerability attributes: threat (psychological, biological, and sociological); barriers (poor access to healthcare, social withdrawal or non-communication, and obstacles from healthcare professionals); and repair in the vulnerability journey during pregnancy and childbirth.^7^ In this context, ‘threat’ represents a potential for harm which is yet to occur, while ‘repair’ refers to the extent to which vulnerability could be minimised through training of healthcare professionals, warm relationships between healthcare providers and women, and women’s empowerment. Colciago et al. (11 studies) categorised vulnerability into deficiency, risk exposure, barriers, and need and mapped 13 vulnerability indices (or characteristics) to these concepts.^15^ Sule et al. (17 studies) reported six vulnerabilities in maternal and newborn health specifically affecting women in LMICs, including restricted access to resources, motherhood limiting economic empowerment, social barriers to healthcare access, reproductive autonomy, power dynamics, and partner support.^16^ de Groot et al. (29 studies), which included only studies from high-income countries, identified the key elements of vulnerability as insufficient material resources, inability to take responsibility for one’s health, unhealthy or risky activity or behaviour, and inadequate social support.^2^ Panel 2 shows an integrative mapping of the vulnerability attributes, indices, and factors reported in these reviews.

*Panel 1* **Summary of methods.**

To identify systematic reviews defining the concept of vulnerability and pregnancy, we conducted comprehensive searches of Medline, EMBASE and APA PsycInfo databases for systematic reviews published between 1 January 1990 and 15 August 2022 (see Appendix 1 for details of the search strategy). There were no language restrictions. These results were used to refine vulnerability framework and identify included vulnerability attributes which formed the terms for further searches (see Appendix 2).

To identify systematic reviews investigating identified vulnerability attributes and pregnancy outcomes and the effectiveness of reparative interventions or strategies on maternal and perinatal complications in low- and middle-income countries (LMICs), we searched Medline and Cochrane databases from 1 January 1990 to 15 August 2022 (see Appendix 2 for details of the search strategies). We used search terms specific for potential vulnerability factors like race and ethnicity, marital status, adolescent pregnancy, parity, gender and sex, nutrition, smoking, alcohol, substance use, environmental disasters, sex work, homelessness, migration status, culture and religion, family violence, socioeconomic status and maternal education. All reviews included involved women from LMICs as per The World Bank definition. Any low-income and middle-income country relevant data presented in studies including high-income countries were extracted. We included reviews of observational and randomised studies presenting quantitative and qualitative data to assess the association between vulnerability attributes and adverse pregnancy outcomes. Reviews of randomised trials were exclusively identified for the evidence on reparative interventions. The PRISMA (Preferred Reporting Items for Systematic Reviews and Meta-Analyses) charts in Appendix 3 provide the details of study identification.

*Panel 2* **Vulnerability attributes affecting women during pregnancy, childbirth, and the postnatal period, as reported in systematic reviews.**

**Table:** 

Vulnerability attributes	Vulnerability indices or factors
**Threat**^a^	
Psychological^a^	Mental health conditions or illness Poor health status or outcome (**Deficiency**^b^)
Biological^a^	Chronic disease Nutritional deficiency Maternal infection Previous caesarean section Primigravida and grandmultiparity Very young or advanced age *Poor health status or outcome (***Deficiency**^b^)
Sociological^a^	Low educational and socioeconomic status (a) Limited economic contributions of women (b) Unstable environment, homelessness Forced marriage Family violence, fear of partner, and unfavourable power dynamics (e) Societal value of women (c) *Socio-demographic characteristics (including age, race and ethnicity, setting)* (**Barrier**^b^) *Risk exposure (including unsafe work environment, war, climate change)* (**Risk**^b^) (3)
**Barrier**^a^	Poor access to healthcare Women withdraw socially or do not communicate with a healthcare provider Feeling stigmatised in pregnancy Lack of companion during pregnancy Negative attitude, perceptions, and engagement of healthcare professionals Lack or varied information about healthcare
	*Distance from care services* (**Deficiency**^b^) (4) *Migration status, language difficulties* (**Barrier**^b^) (2) *Substance abuse* (**Barrier**^b^) (3) *Involvement with the criminal justice system or violence-prone* (**Barrier**^b^) *Homelessness* (**Deficiency**^b^) (1) *Lack of resources* (**Deficiency**^b^) (1) *Care leaver* (**Deficiency**^b^) (4)
**Repair**^a^	Training and education of healthcare professionals Warm relationship between women and professional Individualised flexible care Empowerment of women Supportive partner and family (f) Normalisation of circumstances by removing barriers

de Groot 2019 elements of vulnerability: 1– insufficient material resources; 2– unable to take responsibility for one’s health; 3– unhealthy or risky activity or behaviour; 4– inadequate social support; Sule et al., 2022 vulnerability and resilience dimensions: a—women’s access to resources, b—motherhood limiting women’s economic contributions, c—social barriers to women’s healthcare access, d—women’s reproductive autonomy, e—power dynamics, and f—partner support.

a Briscoe et al., 2016 vulnerability attributes formed the basis of the Table, and other concepts were added where appropriate.

b Colciago et al., 2020 vulnerability concepts and indices (italicised).

**Fig. 1:**
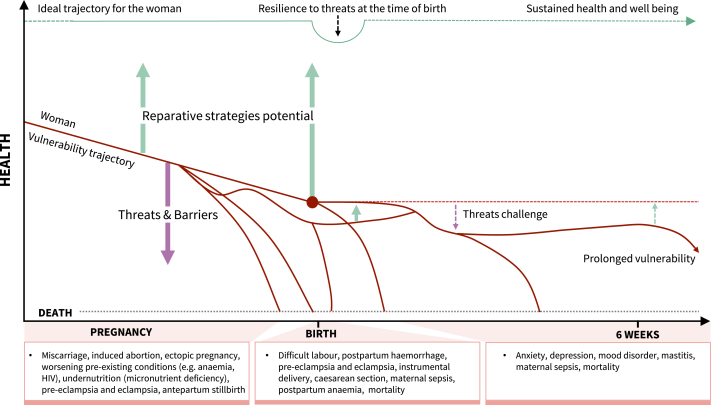
Vulnerability trajectory as a net balance of risk effects (threats), barriers, and protective or recovery factors (reparative strategies). Footnote: figure illustrates typical vulnerability trajectories for pregnant women. Pre-existing threats (including inherent threats due to the pregnancy state) and barriers and weak reparation imply that the woman’s trajectory for health and well-being starts at a suboptimal state. Threats and barriers become cumulative due to repetition or reinforcement as pregnancy advances and are maximal around the time of birth. Adapted from Bill & Melinda Gates Foundation MNCH D&T Growth and Resilience Strategy (with permission).

Two reviews provided conceptual models of vulnerability.^2^^,^^7^ In the model proposed by Briscoe et al., the level of threat increased or decreased according to the degree of the perceived barriers.^7^ The impact of barriers on pregnancy-related outcomes were, in turn, affected by the effectiveness of reparative strategies. Fig. 1 illustrates the push effects of threats and barriers towards adverse outcomes versus the pull effects of reparative strategies in determining a woman’s vulnerability trajectory when pregnancy starts in a less-than-ideal state. This trajectory demonstrates the challenges for reparative interventions in reversing course as risks (or threats) tend to accumulate as pregnancy advances. The conceptual model by de Groot et al. comprised two separate pathways between vulnerability and health—the pathway to adverse outcomes and pathway to health.^2^ The pathway to adverse outcomes involves underlying biological aetiologies like deficiencies and risk exposures, the inability of care pathways to tackle barriers, and ineffective or no reparation. Conversely, the pathway to health or recovery depends on professional and self-care, effective repair, and tackling barriers.^2^ The model acknowledges the asymmetry of these pathways, noting that risks leading to an adverse outcome do not necessarily prevent its recovery and reparative interventions to prevent an adverse outcome do not necessarily improve such outcome once it occurs. It recognises the reinforcement of threats and barriers through the occurrence of an adverse outcome, leading to an even more severe and persistent adverse outcome.

**Fig. 2:**
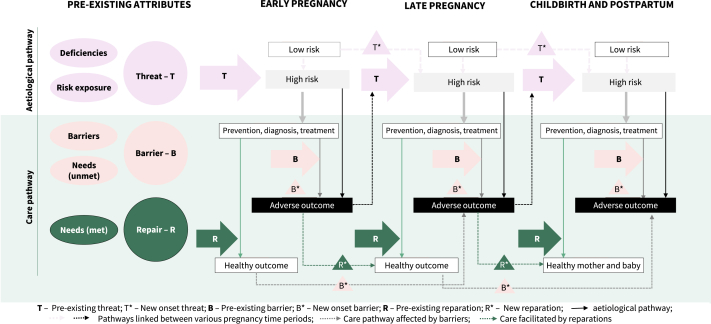
Vulnerability framework through which threats, barriers and reparations influence maternal and perinatal outcomes. Footnote: pregnancies where women have deficiencies or exposure to risks are high-risk pregnancies. Barriers and unmet needs can affect the quality of maternity care, predisposing to adverse outcomes. Effective repair mechanisms in place could prevent adverse outcomes. These vulnerability attributes shown in different colours, Threats, Barriers, and Repair, can influence care and outcome at multiple times during pregnancy through the postpartum continuum. This is illustrated by the arrows interlinking an attribute (T∗/B∗) to healthy or adverse outcomes based on whether an effective reparative strategy (R∗) is involved. Occurrence of an adverse outcome further reinforces threat (T∗) and barrier (B∗) leading to persistence of adverse outcomes during the pregnancy journey. For example, a teenager entering pregnancy with anaemia (T), if diagnosed early in pregnancy and treated (R), could have a healthy outcome. But if she faces barriers in accessing antenatal care because of disempowerment (B), she will likely have complications from worsening anaemia. However, if the high-risk status is recognised in the second or third trimester and reparative interventions are initiated, maternal and perinatal complications are likely to be averted. But, even when anaemia is effectively managed early in pregnancy if new barriers (B∗) arise in later pregnancy (e.g., homelessness), the woman is at increased risk of adverse outcomes (e.g., eclampsia and stillbirth due to lack of antenatal care and undiagnosed pre-eclampsia).

In Fig. 2, we depict a conceptual framework incorporating both models in terms of maternity practice across the pregnancy, childbirth, and postpartum continuum. The presence of a threat (T) can result in a high-risk pregnancy. Barriers (B) to receiving relevant preventative measures, diagnostic tests and/or treatment are likely to contribute to an adverse pregnancy outcome. Conversely, effective reparation (R) can mitigate the risk posed by the barriers, resulting in healthy pregnancy outcomes. The vulnerability attributes could appear, disappear, change, or be reinforced through negative feedback at any point during pregnancy through the postpartum continuum.

In the following sections, we map the available evidence on the magnitude of the association between vulnerability attributes and pregnancy and postpartum complications that contribute to maternal and perinatal mortality and severe morbidity, particularly in LMICs. Table 1 lists vulnerability factors and outcomes reported in existing reviews, categorised as threats and barriers. Supplemental Fig. S1 exemplifies how the underlying threats and barriers are interlinked across early pregnancy, late pregnancy and childbirth.

**Table 1:** Vulnerability attributes associated with adverse outcomes during pregnancy, childbirth, and the postpartum period in systematic reviews (see Appendix 4 for full characteristics of included studies and references).

Vulnerability attribute	Early pregnancy	Late pregnancy	Childbirth	Other outcomes
Threat: deficiencies				
Anaemia		Small for gestational age Low birthweight Preterm birth Pre-eclampsia	Neonatal mortality Postpartum haemorrhage Infection after caesarean section	
Calcium deficiency		Pre-eclampsia		
Socioeconomic status		Stillbirth Preterm birth Low birthweight		
Homelessness	Access to antenatal care	Preterm birth Low birthweight	Birth complications including pre-eclampsia, chorioamnionitis, placental abruption, hypertensive disorders of pregnancy, iron deficiency or other anaemia	
Threat: risk exposure				
Intimate partner violence	Miscarriage Access to antenatal care Termination of pregnancy	Pre-eclampsia Preterm birth Low birthweight Stillbirth	Neonatal mortality Perinatal depression	Postnatal depressive symptoms
Disasters	Access to antenatal care (pandemic)	Stillbirth (pandemic) Pre-eclampsia (terrorist attack) Low birthweight (terrorist attack; natural disasters; wildfires)	Reduced institutional delivery (pandemic) Neonatal mortality (pandemic) Perinatal depression (earthquakes)	Psychosis
Political instability/Fragile and conflict-affected state (FCAS)	Access to antenatal service	Stillbirth Low birthweight		Congenital birth defects
Substance misuse		Low birthweight Preterm birth		
Alcohol		Low birthweight Placenta abruption		
Smoking	Miscarriage	Stillbirth	Neonatal mortality	
Mental health		Low birthweight Preterm birth		
Barriers				
Adolescent pregnancy	Anaemia	Pre-eclampsia Preterm birth Low birthweight	Fetal distress Obstructed labour	
Religion and culture		Low birthweight (fasting mothers) Lower placental weight in fasting mothers	Prolonged labour (Female Genital Mutilation (FGM)) Instrumental delivery (FGM) Postpartum haemorrhage (FGM) Perineal tears (FGM) Fetal distress (FGM) Complications (peripartum hysterectomy; uterine rupture; prolonged labour) associated with faith-based institutions (e.g., spiritual churches)	
Low education	Access to antenatal care Childhood pregnancies Unintended pregnancies	Stillbirth Hypertensive disorders in pregnancy Anaemia	Neonatal mortality Preparation for birth and emergencies	Breastfeeding
Primigravida or grand multiparity		Pre-eclampsia (primigravida) Gestational diabetes	Postpartum haemorrhage Perinatal depression Uterine rupture	
Migrants	Access to antenatal care Miscarriage	Pre-eclampsia Low birthweight Gestational diabetes Small for gestational age Stillbirth Preterm birth	Neonatal mortality Maternal mortality	Infection Caesarean section
Refugees	Miscarriage Access to antenatal service	Stillbirth Preterm birth	Neonatal mortality Maternal death (hypertensive disorders, deep vein thrombosis and pre-eclampsia)	Mental health conditions
Child marriage			Reduced institutional delivery	Intimate partner violence

## Threats and barriers affecting early pregnancy health

The common contributors to maternal mortality and morbidity in early pregnancy are spontaneous miscarriage, ectopic pregnancy, and complications from induced abortion.^17^, ^18^, ^19^ Women also die in early pregnancy from worsening pre-existing medical conditions like anaemia, and infections like malaria and HIV.^20^^,^^21^ The World Health Organization (WHO) recommends first antenatal contact before 12 weeks’ gestation for optimal outcomes.^22^ Early identification of pregnancy and access to antenatal care is essential for commencing preventative and treatment measures for anaemia (e.g., iron supplements), pre-eclampsia (calcium supplements, aspirin), worm infection, malaria (intermittent preventive therapy), and for planning skilled attendance at birth.^22^

We identified nine reviews on threats and 10 reviews on barriers reporting associations with miscarriage, induced abortion, and reduced access to early antenatal care (Table 1). Pregnant women exposed to risky conditions such as intimate partner violence were at increased risk of miscarriage (OR 1.88, 95% CI 1.25–2.82; 1 study),^23^ and induced abortion (OR ranges from 1.10 to 3.80; 5 studies)^24^ and had low antenatal contact in early pregnancy (OR 2.20, 95% CI 1.10–4.40; 1 study) compared to their unexposed counterparts.^25^ Child marriage increased a woman’s risk of experiencing intimate partner violence by her partner by up to four-fold (OR ranges from 1.41 to 4.42; 10 studies).^26^ Moreover, reproductive coercion is a form of violence typically perpetrated by intimate partners or family members.^27^ A qualitative evidence synthesis explained how women experienced reproductive coercion through coerced pregnancies, terminations or contraceptive sabotage, which were driven by social norms around control of women, rigid gender roles, social inequalities and family pressure.^28^

Women living in fragile and conflict-affected states are at high risk of suffering sexual violence and unsafe abortion, the latter a likely consequence of unintended pregnancy.^29^ Poor maternal healthcare service usage in conflict-affected states was associated with adverse maternal and neonatal outcomes, particularly for those individuals with low education and economic status.^30^ Lack of education overall was associated with reduced antenatal care (OR 0.49, 95% CI 0.38–0.63; 18 studies),^31^ a situation worsened by its association with child marriage (OR 2.49, 95% CI 1.58–3.92; 10 studies)^32^ and unintended pregnancies (OR ranges from 1.70 to 6.30; 21 studies).^33^ Pregnant women’s status as refugees and asylum seekers was associated with spontaneous abortion (OR ranges from 1.56 to 2.19; 5 studies) in three reviews.^34^, ^35^, ^36^ In a qualitative review, the key barriers to effective care for pregnant migrant women were their unfamiliarity with the system, limited communication, unmet needs beyond the pregnancy, and lack of respectful care.^37^

Women’s vulnerabilities impact access to early pregnancy tests such as abdominal and pelvic ultrasound,^38^ which is critical to diagnose ectopic pregnancies and incomplete miscarriages. Pregnant adolescents were less likely to have an early ultrasound examination during pregnancy (aOR 0.72, 95% CI 0.59–0.90) than adult women.^39^ Compared to women with no formal education, those with 10 or more years of education were more likely to access early pregnancy ultrasound (aOR 10.3, 95% CI 5.55–19.0).^39^

## Vulnerabilities affecting late pregnancy health

Adverse outcomes in late pregnancy could be from pre-existing vulnerabilities before and in early pregnancy or new threats and barriers manifesting throughout pregnancy (Fig. 2). We identified 18 reviews on deficiencies in maternal health status, 23 on risk exposures, and 18 on barriers to effective care impacting late pregnancy outcomes. Maternal nutritional deficiencies such as calcium deficiency increased the risk of pre-eclampsia (RR 4.06, 95% CI 3.29–7.20).^40^^,^^41^ Stratified analysis showed maternal anaemia was associated in LMICs with preterm birth (OR 4.07, 95% CI 1.92–8.65; 17 studies),^42^ low birthweight (OR 2.17, 95% CI 1.63–2.90; 24 studies),^42^ and, in sub-Saharan Africa, pre-eclampsia (OR 3.22, 95% CI 2.70–3.75; 4 studies).^43^^,^^44^

The main barriers to the uptake of recommended nutrient supplementation in pregnancy, such as calcium, are women’s lack of knowledge about pre-eclampsia and concerns about taking supplements during pregnancy^45^; healthcare professionals’ beliefs about the intervention, their knowledge about the condition and external structural factors including workload, stockout and lack of equipment. Three reviews^43^^,^^44^^,^^46^ reported the association between maternal characteristics such as nulliparity and pre-eclampsia (OR 2.52, 95% CI 1.19–3.86; 6 studies), which could be attributed to either the biological pathway^47^ or care pathway with delayed or no risk assessment.^48^ Two reviews^49^^,^^50^ reported that women with perinatal depression were at increased risk of low birthweight babies (RR 1.66, 95% CI 1.06–2.61; 6 studies) and preterm birth (RR 2.68, 95% CI 1.89–2.92; 4 studies) compared to those without a mental health condition.

Maternal exposure to risks and risky behaviours directly and indirectly contributes to pregnancy complications. Maternal smoking was associated with an increased risk of stillbirth (OR 1.60, 95% CI 1.23–2.08; 1 study).^51^ Alcohol intake was variously associated with low birthweight (OR ranges from 1.62 to 4.20; 20 studies),^52^ and placental abruption (OR 1.48, 95% CI 1.37–1.60; 4 studies).^53^ In a review of studies from Latin America, women exposed to intimate partner violence during pregnancy were at increased risk of pre-eclampsia (OR 2.70, 95% CI 1.90–3.90; 1 study) and were more likely to have low birthweight (OR ranges from 3.30 to 4.00; 5 studies) or stillborn babies (OR ranges from 1.43 to 1.65; 2 studies) than unexposed women.^23^ Women exposed to intimate partner or family violence attributed depression and stress to not initiating antenatal care (OR 2.20, 95% CI 1.10–4.40; 1 study) and subsequently had fewer antenatal visits.^23^ They may not always disclose experiences of violence for fear of reprisals, embarrassment and shame.^54^ Lack of regular antenatal care in late pregnancy could result in missing early diagnosis of pre-eclampsia and small-for-gestational-age babies.

Eighteen reviews reported age, education, socioeconomic status, and internal and external displacements as barriers to effective antenatal care, and their associated complications. Adolescents in sub-Saharan Africa were at higher risk of pre-eclampsia (OR 3.52, 95% CI 2.26–5.48; 3 studies) compared to adult women.^55^ Lower educational status was consistently associated with poor use of maternal health services and antenatal care^56^^,^^57^ and lack of formal education was associated with term stillbirth (OR ranges from 2.30 to 8.50; 2 studies).^58^ Conversely, a protective effect was observed for maternal education and having a low birthweight baby (OR 0.67, 95% CI 0.61–0.74; 35 studies),^59^ and women with secondary school education were less likely to have a small-for-gestational-age baby (RR 0.87, 95% CI 0.77–0.98; 1 study) than less educated women.^60^ Women experiencing poverty were more likely to experience stillbirth (3 studies),^58^ preterm birth (rural setting: RR 1.49, 95% CI 1.32–1.68; 1 study; urban setting: RR 1.21, 95% CI 1.00–1.46; 1 study), and low birthweight (OR 2.10, 95% CI 1.42–3.05; 1 study) than affluent women.^60^

Women from refugee backgrounds had higher rates of stillbirth (OR/RR ranges from 1.20 to 12.29; 10 studies)^35^^,^^36^^,^^61^ and preterm birth (OR ranges from 0.72 to 1.28; 5 studies)^35^^,^^36^^,^^62^ than host country women in four reviews. Maternal deaths were higher in migrant versus host country populations in one review, mainly from hypertensive disorders and venous thromboembolism—conditions that are preventable through access to timely and effective care.^35^ Non-refugee migrants from non-European countries were at higher risk of stillbirth (RR 1.88, 95% CI 1.58–2.23; 5 studies) than European non-refugee migrants in five studies, indicative of potential racial and ethnic disparities in care.^61^ Adverse environmental settings, directly and indirectly, impacted pregnancies through reduced antenatal care. The rates of low birthweight babies were increased in women exposed to wildfires, terrorist attacks^63^ and those living in fragile and conflict-affected states.^64^ Stillbirth rates were increased in women exposed to chemical warfare, likely from direct impact.^64^

## Vulnerability attributes and childbirth

We identified 10 reviews on threats and 19 reviews on barriers that women face during childbirth (Table 1). In addition to the risk of experiencing potentially life-threatening complications, women are most vulnerable to physical, verbal, emotional abuse and mistreatment, and loss of autonomy in decision-making during this period.^65^ During childbirth, women face threats from pre-existing deficiencies such as nutritional anaemia. Prenatal anaemia was associated with increased risks of postpartum haemorrhage (OR 3.54, 95% CI 1.20–10.4; 5 studies)^66^ and surgical site infection (OR 4.56, 95% CI 2.88–7.22; 4 studies).^67^ Maternal obstetric characteristics such as grandmultiparity was associated with an increased risk of postpartum haemorrhage (OR 6.58, 95% CI 1.90–22.80; 2 studies).^68^

Sociocultural practices, beliefs, and norms dictating women-specific practices pose threats and barriers to childbirth. In a systematic review of studies from Nigeria, three-quarters of women requiring peripartum hysterectomies had previously attended spiritual churches in labour, and 40% of asphyxiated babies were born in faith-based institutions.^69^ Four reviews^70^, ^71^, ^72^, ^73^ on women exposed to harmful cultural practices like female genital mutilation showed that such women were at increased risk of prolonged labour (OR 1.49, 95% CI 1.01–2.19; 4 studies), perineal tear (RR 2.15, 95% CI 1.08–4.27; 5 studies), and postpartum haemorrhage (OR 1.50, 95% CI 1.22–1.84; 5 studies; RR 2.59, 95% CI 1.28–5.25; 2 studies) especially when giving birth in settings not specialised in caring for women with female genital mutilation. Systematic reviews reported child-mothers in LMICs were less likely to give birth in a healthcare facility or have a skilled healthcare provider during birth compared to those married as adults (OR ranges from 0.52 to 0.79; 7 studies).^26^

Health emergencies resulting from conflicts, pathogenic outbreaks like COVID-19, and aftermath of climate events pose significant threats to maternal health services, particularly around the time of birth, when the risk of mortality and morbidity is greatest for the woman and her baby. During epidemics and pandemics of the last decade, women’s access to childbirth care in LMICs were significantly reduced. Facility-based birth rates were reduced by up to half during the COVID-19 pandemic in India and Nepal and by 20% during the Ebola epidemic in rural Guinea, and higher rates of adverse maternal and perinatal outcomes were reported.^74^ Migration and displacement are associated with increased perinatal mortality. Higher perinatal mortality rates have been reported in pregnant registered refugees (RR 1.71, 95% CI 1.41–2.06; 5 studies) and non-refugee migrants from non-European countries (RR 1.54, 95% CI 1.39–1.69; 5 studies) compared with the host country population.^61^

Even when potentially life-saving drugs and procedures are available, their substandard status contributes to poor outcomes. In a systematic review assessing the quality of maternal medicines, 49% of uterotonic drugs used for managing postpartum haemorrhage (19 studies) and 13% of injectable antibiotics for managing sepsis in LMICs failed quality tests (3 studies).^75^ Maternal death rates were 100-fold higher in women undergoing a caesarean section, a potentially life-saving procedure, in LMICs than in high-income countries.^76^

In LMICs, facility-based births are promoted to improve childbirth outcomes.^77^ However, women continue to be exposed to physical abuse, verbal and emotional abuse, and discrimination when giving birth in health facilities.^65^ In a systematic review, 44% (95% CI 29.9–58.2; 34 studies) experienced disrespect and abuse during childbirth at health facilities in sub-Saharan Africa; 16% (95% CI 13.4–18.2; 29 studies) physical abuse, 17% (95% CI 14.5–19.2; 28 studies) non-confidential care and 17% (95% CI 13.9–19.8; 30 studies) abandonment.^78^ Women may, therefore, prefer to give birth at home or in the community to avoid this mistreatment, which may place them at risk of obstetric complications.^79^

## Reparation of vulnerabilities during pregnancy, childbirth, and the postpartum period

Reparative strategies that tackle various vulnerability factors impinging on maternal and perinatal health require a life course approach, as these need to address the interlinking factors affecting the woman's health pre-pregnancy, during pregnancy, and beyond. Appendix 5 lists the interventions reported in systematic reviews of randomised trials to address maternal vulnerabilities.

Optimising maternal health by addressing nutritional deficiencies like iron and calcium deficiencies effectively improves pregnancy and childbirth outcomes. In three systematic reviews, iron supplementation is effective in reducing the risk of maternal anaemia at term (RR 0.30, 95% CI 0.19–0.46; 14 studies), iron deficiency at term (RR 0.43, 95% CI 0.27–0.66; 7 studies) and having a low birthweight baby (RR 0.81, 95% CI 0.68–0.97; 11 studies).^80^, ^81^, ^82^ When taken as recommended, antenatal iron supplementation can prevent 20% of maternal deaths and halve neonatal mortality.^83^^,^^84^ Calcium supplementation in calcium-deficient populations reduces the risk of pre-eclampsia (RR 0.45, 95% CI 0.31–0.65; 13 studies) and reduces severe morbidity and mortality by 20% (RR 0.80, 95% CI 0.66–0.98; 4 studies).^85^ While reparation of the underlying deficiency state effectively prevents anaemia-related complications and pre-eclampsia, we did not find any systematic reviews on the effects of implementation strategies to improve uptake of these interventions. A qualitative evidence synthesis identified facilitators for increasing uptake of calcium supplementation, including women receiving information regarding pre-eclampsia and safety of supplement use from reliable sources, having alternative dosing options and reminders to take supplements, and support from families and communities (Cormick G, unpublished). For healthcare providers, training, adequate staffing, and reliable calcium supply promoted calcium use for pregnant women.^86^

The effects of interventions to improve pregnancy-related outcomes in women exposed to risks or risky behaviours were reported in 33 reviews. A Cochrane review of interventions to minimise the harmful impact of smoking in pregnancy included four trials from Latin America.^87^ Psychosocial support in the form of counselling increased the rate of smoking cessation in late pregnancy (RR 1.83, 95% CI 1.22–2.73; 1 study) compared to usual care or less intensive intervention.^87^ Of the nine trials in a Cochrane review that examined interventions to minimise adverse outcomes from intimate partner violence, only two were conducted in LMICs (China, Peru).^88^, ^89^, ^90^ An intervention tailored to improve independence and autonomy in Chinese mothers appeared to minimise postnatal depression (RR 0.39, 95% CI 0.20–0.75).^90^ The other trial in Peruvian women focused on encouraging safety planning and behaviours which reduced abuse (RR 2.60, 95% CI 1.41–4.79).^88^^,^^89^ Eight reviews reported psychological interventions to minimise the risk of perinatal depression.^91^, ^92^, ^93^, ^94^, ^95^, ^96^, ^97^, ^98^ A review of randomised controlled trials demonstrated mindfulness-based interventions reduced perinatal depressive (standardized mean difference −0.77, −1.09 to −0.44; 22 studies) and perinatal anxiety symptoms (Standard mean difference −1.29, −2.09 to −0.49; 17 studies) compared to controls.^92^

Interventions to minimise the impact of barriers women face during pregnancy and childbirth, including access to healthcare, were reported in 19 reviews. In the two systematic reviews reporting on interventions to tackle adolescent pregnancy as a maternal health risk, school-based programmes, health counselling, and cash transfers have effectively reduced adolescent pregnancies and improved pregnancy outcomes.^56^^,^^99^ Education of adolescent mothers appeared to be the significant predictor of use of maternal health services in a review of observational studies.^57^ In a Cochrane review, community health educational interventions such as one-to-one or group counselling significantly reduced neonatal mortality (RR 0.87, 95% CI 0.78–0.96; 26 studies) and perinatal mortality (RR 0.83, 95% CI 0.75–0.91; 15 studies), improved the use of any antenatal care (RR 1.16, 95% CI 1.11–1.22; 18 studies), and increased initiation of breastfeeding (RR 1.56, 95% CI 1.37–1.77; 19 studies).^100^ Nine reviews reported on interventions to minimise the risk of unintended pregnancy among adolescents.^56^^,^^57^^,^^99^^,^^101^, ^102^, ^103^, ^104^, ^105^, ^106^ The combination of educational and contraceptive-promoting interventions significantly lowered the risk of unintended pregnancy among adolescents (RR 0.66, 95% CI 0.50–0.87; 4 studies).^104^ A Cochrane review focusing on community-based interventions showed improvements in antenatal care coverage (OR 1.11, 1.01–1.22; 10 studies) but no effect on maternal or perinatal outcomes.^107^ Alongside this, financial incentives through conditional cash transfers have been shown to be effective in improving antenatal care attendance^108^; in Kenya, this intervention encouraged a continuum of care throughout pregnancy and the postnatal period (aOR 1.90, 1.36–2.66).^109^

## Call for policymakers, healthcare professionals, and researchers to address key threats and barriers to antenatal, childbirth, and postpartum care

The interdependency and dynamic nature of vulnerability attributes demands a holistic approach to address vulnerabilities underpinning poor pregnancy outcomes. To improve and sustain women’s health and wellbeing, a life course approach is required to tackle vulnerability attributes in national policies, public health and health care settings, and research, in line with the third and fifth goals of the 2030 agenda for sustainable development.^110^ Policymakers, healthcare professionals, and researchers have moral and ethical obligations to reduce threats, remove barriers, and foster reparations along the pregnancy journey and beyond.

Policymakers should use the concept of vulnerability to justify access to increased resources for maternal health at the country level based on a careful assessment of prevailing threats and barriers that impact pregnancy outcomes within their local contexts. The goal should be to apply the concept to create just and equitable health systems that promote autonomy and support wellbeing for all rather than serve as a marker of susceptibility that widens social control or leads to exclusion, stigmatisation, or disempowerment of any disadvantaged groups.^111^^,^^112^ As many vulnerability factors and indices often co-exist, addressing broader determinants of maternal health at a policy level is paramount. Policymakers should prioritise women’s access to education and empowerment through training programmes to enter the workforce, as these can significantly impact maternal and perinatal health. Education and empowerment of women are key reparative strategies to advance maternal health through better understanding of their own health issues, avoiding pregnancies at a young age, improving access to and use of contraception, seeking antenatal care early, and improved decision-making. Effective community and health systems investment is crucial in addressing vulnerabilities holistically. Investment in contraceptive uptake programmes can be effective in avoiding multiple vulnerability and preventing the prolongation of vulnerability across pregnancies in the same woman.^113^^,^^114^ Ensuring women can access various contraceptive methods is critical for them to space their pregnancies and prevent unintended pregnancies, thereby reducing unsafe abortions and high-risk births while simultaneously facilitating women’s bodily autonomy and choice and reducing gender inequality.^110^

Healthcare professionals caring for pregnant women have an important role in identifying and addressing vulnerabilities in maternity care. They should ensure that the woman’s vulnerability trajectory along the pregnancy journey is captured in care plans and register any pre-existing and potential threats, barriers, and corresponding interventions to build resilience and optimise pregnancy outcomes. Locally validated tools (e.g., checklist, questionnaire) can be introduced to identify vulnerability points towards developing individual and targeted coping strategies to support women at risk of adverse pregnancy outcomes. The approach to care should embrace the principles of growth and resilience, shifting from syndromic management to risk assessment and interventions in the early part of the pregnancy journey and prioritising prevention over treatment. It is crucial for healthcare professionals to effectively communicate women’s personal vulnerabilities, as well as opportunities and limitations within the health system so that care can be tailored appropriately. Care providers should be aware that women with multiple vulnerabilities are less likely to receive the care they need,^115^ and mapping additional care within existing services is a critical step for improving the organisation of maternity services and maternal and perinatal outcomes.

Researchers should focus on obtaining robust, standardised data to identify the various vulnerability factors and their interactions in order to plan targeted interventions that are specific to the woman and the setting. For example, not enough is known about the impact of internal and external displacement of women due to adverse environmental factors, including war, natural disasters, and climate change on pregnancy outcomes. Such research should aim to provide a better understanding of the underlying causes of disparities in maternal and perinatal health outcomes across settings. Given the paucity of evidence on reparative strategies versus the long list of threats and barriers identified in our review, studies on the best strategies to scale proven interventions for addressing leading causes of maternal mortality and morbidity should be top priority. High-quality evaluations of programmes, including their economic impact, should be conducted to scale up evidence-based recommendations at local, regional, and national levels.

Another major research gap is how to identify and respond to the root causes of mistreatment during childbirth in order to transform health systems and improve health workers' practices. Most importantly, women’s voices should be central to all efforts to address vulnerability and improve pregnancy outcomes. It is essential to understand the perception of individual pregnant women about their own vulnerability as this could provide valuable information in developing appropriate reparative strategies. Women and families with vulnerabilities should be directly involved in prioritising and designing research and policy to ensure that these are relevant to the actual needs and limitations experienced by people in the real world.

## Contributors

ST and OTO conceptualised the study. JS, JA, TK and HK were involved in data curation. JS and JA analysed the data. All authors interpreted the results. JS and JA are joint first authors. OTO and ST are joint last authors. All co-authors had full access to data in the study, contributed to the writing of the manuscript and approved the final version. ST, OTO and JA are the guarantors and verified the data in the study. All co-authors accept responsibility for the decision to submit for publication. The corresponding author attests that all listed authors meet authorship criteria and that no others meeting the criteria have been omitted.

## Data sharing statement

All data requests should be submitted to the corresponding author for consideration.

## Declaration of interests

All authors have completed the ICMJE uniform disclosure form at www.icmje.org/coi_disclosure.pdf and declare no financial relationships with any organisations that might have an interest in the submitted work in the previous three years; no other relationships or activities that could appear to have influenced the submitted work. The contents of the paper are the sole responsibility of the authors and do not necessarily represent the official views, decisions, or policies of HRP, World Health Organization or other authors’ organisations.

## Appendix ASupplementary data

Appendices 1–5 and Supplementary Fig. S1

## References

[1] Scheele J., Harmsen van der Vliet–Torij H.W., Wingelaar-Loomans E.M., Goumans M.J.B.M. (2020). Defining vulnerability in European pregnant women, a Delphi study. Midwifery.

[2] de Groot N., Bonsel G.J., Birnie E., Valentine N.B. (2019). Towards a universal concept of vulnerability: broadening the evidence from the elderly to perinatal health using a Delphi approach. PLoS One.

[3] World Health Organization (2019). https://apps.who.int/iris/handle/10665/327595.

[4] Simms C.D., Persaud D.D. (2009). Global health and local poverty: rich countries’ responses to vulnerable populations. Can J Public Health.

[5] Waisel D.B. (2013). Vulnerable populations in healthcare. Curr Opin Anesthesiol.

[6] Gaynor T.S., Wilson M.E. (2020). Social vulnerability and equity: the disproportionate impact of COVID-19. Public Adm Rev.

[7] Briscoe L., Lavender T., McGowan L. (2016). A concept analysis of women's vulnerability during pregnancy, birth and the postnatal period. J Adv Nurs.

[8] Hussein J. (2017). Non-communicable diseases during pregnancy in low and middle income countries. Obstet Med.

[9] World Health Organization (2022). Maternal mortality. https://www.who.int/news-room/fact-sheets/detail/maternal-mortality.

[10] Ashorn P., Ashorn U., Muthiani Y. (2023). Small vulnerable newborns-big potential for impact. Lancet.

[11] Lawn J.E., Ohuma E.O., Bradley E. (2023). Small babies, big risks: global estimates of prevalence and mortality for vulnerable newborns to accelerate change and improve counting. Lancet.

[12] Hofmeyr G.J., Black R.E., Rogozińska E. (2023). Evidence-based antenatal interventions to reduce the incidence of small vulnerable newborns and their associated poor outcomes. Lancet.

[13] Phipps E.A., Thadhani R., Benzing T., Karumanchi S.A. (2019). Pre-eclampsia: pathogenesis, novel diagnostics and therapies. Nat Rev Nephrol.

[14] Grote N.K., Bridge J.A., Gavin A.R., Melville J.L., Iyengar S., Katon W.J. (2010). A meta-analysis of depression during pregnancy and the risk of preterm birth, low birth weight, and intrauterine growth restriction. Arch Gen Psychiatry.

[15] Colciago E., Merazzi B., Panzeri M., Fumagalli S., Nespoli A. (2020). Women's vulnerability within the childbearing continuum: a scoping review. Eur J Midwifery.

[16] Sule F.A., Uthman O.A., Olamijuwon E.O. (2022). Examining vulnerability and resilience in maternal, newborn and child health through a gender lens in low-income and middle-income countries: a scoping review. BMJ Glob Health.

[17] Say L., Chou D., Gemmill A. (2014). Global causes of maternal death: a WHO systematic analysis. Lancet Glob Health.

[18] Bearak J., Popinchalk A., Ganatra B. (2020). Unintended pregnancy and abortion by income, region, and the legal status of abortion: estimates from a comprehensive model for 1990–2019. Lancet Glob Health.

[19] Ganatra B., Gerdts C., Rossier C. (2017). Global, regional, and subregional classification of abortions by safety, 2010–14: estimates from a Bayesian hierarchical model. Lancet.

[20] Bone J.N., Bellad M., Goudar S. (2022). Anemia and adverse outcomes in pregnancy: subgroup analysis of the CLIP cluster-randomized trial in India. BMC Pregnancy Childbirth.

[21] Ssentongo P., Ba D.M., Ssentongo A.E. (2020). Associations of malaria, HIV, and coinfection, with anemia in pregnancy in sub-Saharan Africa: a population-based cross-sectional study. BMC Pregnancy Childbirth.

[22] World Health Organization (2017). https://www.who.int/publications/i/item/9789241549912.

[23] Han A., Stewart D.E. (2013). Maternal and fetal outcomes of intimate partner violence associated with pregnancy in the Latin American and Caribbean region. Int J Gynecol Obstet.

[24] Hall M., Chappell L.C., Parnell B.L., Seed P.T., Bewley S. (2014). Associations between intimate partner violence and termination of pregnancy: a systematic review and meta-analysis. PLoS Med.

[25] Moraes C.L., Arana F.D.N., Reichenheim M.E. (2010). Violência física entre parceiros íntimos na gestação como fator de risco para a má qualidade do pré-natal. Rev Saude Publica.

[26] Fan S., Koski A. (2022). The health consequences of child marriage: a systematic review of the evidence. BMC Public Health.

[27] Tarzia L., Hegarty K. (2021). A conceptual re-evaluation of reproductive coercion: centring intent, fear and control. Reprod Health.

[28] Moulton J.E., Corona M.I.V., Vaughan C., Bohren M.A. (2021). Women's perceptions and experiences of reproductive coercion and abuse: a qualitative evidence synthesis. PLoS One.

[29] Ba I., Bhopal R.S. (2017). Physical, mental and social consequences in civilians who have experienced war-related sexual violence: a systematic review (1981–2014). Public Health.

[30] Gopalan S.S., Das A., Howard N. (2017). Maternal and neonatal service usage and determinants in fragile and conflict-affected situations: a systematic review of Asia and the Middle-East. BMC Wom Health.

[31] Tesfaye G., Loxton D., Chojenta C., Semahegn A., Smith R. (2017). Delayed initiation of antenatal care and associated factors in Ethiopia: a systematic review and meta-analysis. Reprod Health.

[32] Kassa G.M., Arowojolu A.O., Odukogbe A.A., Yalew A.W. (2018). Prevalence and determinants of adolescent pregnancy in Africa: a systematic review and Meta-analysis. Reprod Health.

[33] Alene M., Yismaw L., Berelie Y., Kassie B., Yeshambel R., Assemie M.A. (2020). Prevalence and determinants of unintended pregnancy in Ethiopia: a systematic review and meta-analysis of observational studies. PLoS One.

[34] Almeida L.M., Caldas J., Ayres-de-Campos D., Salcedo-Barrientos D., Dias S. (2013). Maternal healthcare in migrants: a systematic review. Matern Child Health J.

[35] Heslehurst N., Brown H., Pemu A., Coleman H., Rankin J. (2018). Perinatal health outcomes and care among asylum seekers and refugees: a systematic review of systematic reviews. BMC Med.

[36] Harakow H., Hvidman L., Wejse C., Eiset A.H. (2021). Pregnancy complications among refugee women: a systematic review. Acta Obstet Gynecol Scand.

[37] Fair F., Raben L., Watson H. (2020). Migrant women's experiences of pregnancy, childbirth and maternity care in European countries: a systematic review. PLoS One.

[38] World Health Organization (2022). https://www.who.int/publications-detail-redirect/9789240046009.

[39] Kozuki N., Katz J., Khatry S.K., Tielsch J.M., LeClerq S.C., Mullany L.C. (2016). Community survey on awareness and use of obstetric ultrasonography in rural Sarlahi District, Nepal. Int J Gynaecol Obstet.

[40] Cormick G., Betrán A.P., Romero I.B. (2019). Global inequities in dietary calcium intake during pregnancy: a systematic review and meta-analysis. BJOG.

[41] Segovia B.L., Vega I.T., Villarreal E.C., Licona N.A. (2004). [Hypocalciuria during pregnancy as a risk factor of preeclampsia]. Ginecol Obstet Mex.

[42] Jung J., Rahman M.M., Rahman M.S. (2019). Effects of hemoglobin levels during pregnancy on adverse maternal and infant outcomes: a systematic review and meta-analysis. Ann N Y Acad Sci.

[43] Meazaw M.W., Chojenta C., Muluneh M.D., Loxton D. (2020). Factors associated with hypertensive disorders of pregnancy in sub-Saharan Africa: a systematic and meta-analysis. PLoS One.

[44] Meazaw M.W., Chojenta C., Muluneh M.D., Loxton D. (2020). Systematic and meta-analysis of factors associated with preeclampsia and eclampsia in sub-Saharan Africa. PLoS One.

[45] World Health Organization (2018). https://apps.who.int/iris/handle/10665/277235.

[46] Luo Z.C., An N., Xu H.R., Larante A., Audibert F., Fraser W.D. (2007). The effects and mechanisms of primiparity on the risk of pre-eclampsia: a systematic review. Paediatr Perinat Epidemiol.

[47] Bdolah Y., Elchalal U., Natanson-Yaron S. (2014). Relationship between nulliparity and preeclampsia may be explained by altered circulating soluble fms-like tyrosine kinase 1. Hypertens Pregnancy.

[48] Salam R.A., Das J.K., Ali A., Bhaumik S., Lassi Z.S. (2015). Diagnosis and management of preeclampsia in community settings in low and middle-income countries. J Fam Med Prim Care.

[49] Dadi A.F., Miller E.R., Mwanri L. (2020). Antenatal depression and its association with adverse birth outcomes in low and middle-income countries: a systematic review and meta-analysis. PLoS One.

[50] Dadi A.F., Miller E.R., Bisetegn T.A., Mwanri L. (2020). Global burden of antenatal depression and its association with adverse birth outcomes: an umbrella review. BMC Public Health.

[51] Marufu T.C., Ahankari A., Coleman T., Lewis S. (2015). Maternal smoking and the risk of still birth: systematic review and meta-analysis. BMC Public Health.

[52] Pereira P.P.D.S., Mata F.A.F.D., Figueiredo A.C.M.G., Silva R.B., Pereira M.G. (2019). Maternal exposure to alcohol and low birthweight: a systematic review and meta-analysis. Rev Bras Ginecol Obstet.

[53] Steane S.E., Young S.L., Clifton V.L., Gallo L.A., Akison L.K., Moritz K.M. (2021). Prenatal alcohol consumption and placental outcomes: a systematic review and meta-analysis of clinical studies. Am J Obstet Gynecol.

[54] Amel Barez M., Mirzaii Najmabadi K., Latifnejad Roudsari R., Mousavi Bazaz M., Babazadeh R. (2022). ‘It is a hard decision’: a qualitative study of perinatal intimate partner violence disclosure. Reprod Health.

[55] Grønvik T., Fossgard Sandøy I. (2018). Complications associated with adolescent childbearing in Sub-Saharan Africa: a systematic literature review and meta-analysis. PLoS One.

[56] Banke-Thomas O.E., Banke-Thomas A.O., Ameh C.A. (2017). Factors influencing utilisation of maternal health services by adolescent mothers in Low-and middle-income countries: a systematic review. BMC Pregnancy Childbirth.

[57] Mekonnen T., Dune T., Perz J. (2019). Maternal health service utilisation of adolescent women in sub-Saharan Africa: a systematic scoping review. BMC Pregnancy Childbirth.

[58] Di Mario S., Say L., Lincetto O. (2007). Risk factors for stillbirth in developing countries: a systematic review of the literature. Sex Transm Dis.

[59] Godah M.W., Beydoun Z., Abdul-Khalek R.A., Safieddine B., Khamis A.M., Abdulrahim S. (2021). Maternal education and low birth weight in low- and middle-income countries: systematic review and meta-analysis. Matern Child Health J.

[60] Ngandu C.B., Momberg D., Magan A., Chola L., Norris S.A., Said-Mohamed R. (2020). The association between household socio-economic status, maternal socio-demographic characteristics and adverse birth and infant growth outcomes in sub-Saharan Africa: a systematic review. J Dev Orig Health Dis.

[61] Gissler M., Alexander S., MacFarlane A. (2009). Stillbirths and infant deaths among migrants in industrialized countries. Acta Obstet Gynecol Scand.

[62] Gagnon A.J., Zimbeck M., Zeitlin J. (2009). Migration to western industrialised countries and perinatal health: a systematic review. Soc Sci Med.

[63] Harville E., Xiong X., Buekens P. (2010). Disasters and perinatal health: a systematic review. Obstet Gynecol Surv.

[64] Keasley J., Blickwedel J., Quenby S. (2017). Adverse effects of exposure to armed conflict on pregnancy: a systematic review. BMJ Glob Health.

[65] Bohren M.A., Mehrtash H., Fawole B. (2019). How women are treated during facility-based childbirth in four countries: a cross-sectional study with labour observations and community-based surveys. Lancet.

[66] Omotayo M.O., Abioye A.I., Kuyebi M., Eke A.C. (2021). Prenatal anemia and postpartum hemorrhage risk: a systematic review and meta-analysis. J Obstet Gynaecol Res.

[67] Getaneh T., Negesse A., Dessie G. (2020). Prevalence of surgical site infection and its associated factors after cesarean section in Ethiopia: systematic review and meta-analysis. BMC Pregnancy Childbirth.

[68] Nigussie J., Girma B., Molla A., Tamir T., Tilahun R. (2022). Magnitude of postpartum hemorrhage and its associated factors in Ethiopia: a systematic review and meta-analysis. Reprod Health.

[69] Hirose A., Owolabi O., Imamura M., Okonofua F., Hussein J. (2017). Systematic review of obstetric care from a women-centered perspective in Nigeria since 2000. Int J Gynecol Obstet.

[70] Obermeyer C.M. (2005). The consequences of female circumcision for health and sexuality: an update on the evidence. Cult Health Sex.

[71] Berg R.C., Underland V., Odgaard-Jensen J., Fretheim A., Vist G.E. (2014). Effects of female genital cutting on physical health outcomes: a systematic review and meta-analysis. BMJ Open.

[72] Lurie J.M., Weidman A., Huynh S., Delgado D., Easthausen I., Kaur G. (2020). Painful gynecologic and obstetric complications of female genital mutilation/cutting: a systematic review and meta-analysis. PLoS Med.

[73] Sylla F., Moreau C., Andro A. (2020). A systematic review and meta-analysis of the consequences of female genital mutilation on maternal and perinatal health outcomes in European and African countries. BMJ Glob Health.

[74] Palo S.K., Dubey S., Negi S. (2022). Effective interventions to ensure MCH (Maternal and Child Health) services during pandemic related health emergencies (Zika, Ebola, and COVID-19): a systematic review. PLoS One.

[75] Torloni M.R., Bonet M., Betrán A.P., Ribeiro-do-Valle C.C., Widmer M. (2020). Quality of medicines for life-threatening pregnancy complications in low- and middle-income countries: a systematic review. PLoS One.

[76] Sobhy S., Arroyo-Manzano D., Murugesu N. (2019). Maternal and perinatal mortality and complications associated with caesarean section in low-income and middle-income countries: a systematic review and meta-analysis. Lancet.

[77] Bohren M.A., Hunter E.C., Munthe-Kaas H.M., Souza J.P., Vogel J.P., Gülmezoglu A.M. (2014). Facilitators and barriers to facility-based delivery in low- and middle-income countries: a qualitative evidence synthesis. Reprod Health.

[78] Kassa Z.Y., Tsegaye B., Abeje A. (2020). Disrespect and abuse of women during the process of childbirth at health facilities in sub-Saharan Africa: a systematic review and meta-analysis. BMC Int Health Hum Rights.

[79] Bohren M.A., Vogel J.P., Hunter E.C. (2015). The mistreatment of women during childbirth in health facilities globally: a mixed-methods systematic review. PLoS Med.

[80] Peña-Rosas J.P., De-Regil L.M., Garcia-Casal M.N., Dowswell T. (2015). Daily oral iron supplementation during pregnancy. Cochrane Database Syst Rev.

[81] Peña-Rosas J.P., De-Regil L.M., Dowswell T., Viteri F.E. (2012). Daily oral iron supplementation during pregnancy. Cochrane Database Syst Rev.

[82] Lewkowitz A.K., Gupta A., Simon L. (2019). Intravenous compared with oral iron for the treatment of iron-deficiency anemia in pregnancy: a systematic review and meta-analysis. J Perinatol.

[83] Bhutta Z.A., Ahmed T., Black R.E. (2008). What works? Interventions for maternal and child undernutrition and survival. Lancet.

[84] Njiru H., Njogu E., Gitahi M.W., Kabiru E. (2022). Effectiveness of public health education on the uptake of iron and folic acid supplements among pregnant women: a stepped wedge cluster randomised trial. BMJ Open.

[85] Hofmeyr G.J., Lawrie T.A., Atallah Á.N., Torloni M.R. (2018). Calcium supplementation during pregnancy for preventing hypertensive disorders and related problems. Cochrane Database Syst Rev.

[86] Omotayo M.O., Dickin K.L., O’Brien K.O., Neufeld L.M., De Regil L.M., Stoltzfus R.J. (2016). Calcium supplementation to prevent preeclampsia: translating guidelines into practice in low-income countries. Adv Nutr.

[87] Chamberlain C., O’Mara-Eves A., Porter J. (2017). Psychosocial interventions for supporting women to stop smoking in pregnancy. Cochrane Database Syst Rev.

[88] Jahanfar S., Howard L.M., Medley N. (2014). Interventions for preventing or reducing domestic violence against pregnant women. Cochrane Database Syst Rev.

[89] Cripe S.M., Sanchez S.E., Sanchez E. (2010). Intimate partner violence during pregnancy: a pilot intervention program in Lima, Peru. J Interpers Violence.

[90] Tiwari A., Leung W.C., Leung T.W., Humphreys J., Parker B., Ho P.C. (2005). A randomised controlled trial of empowerment training for Chinese abused pregnant women in Hong Kong. BJOG.

[91] Chowdhary N., Sikander S., Atif N. (2014). The content and delivery of psychological interventions for perinatal depression by non-specialist health workers in low and middle income countries: a systematic review. Best Pract Res Clin Obstet Gynaecol.

[92] Leng L.L., Yin X.C., Ng S.M. (2023). Mindfulness-based intervention for clinical and subthreshold perinatal depression and anxiety: a systematic review and meta-analysis of randomized controlled trial. Compr Psychiatry.

[93] Munodawafa M., Mall S., Lund C., Schneider M. (2018). Process evaluations of task sharing interventions for perinatal depression in low and middle income countries (LMIC): a systematic review and qualitative meta-synthesis. BMC Health Serv Res.

[94] Dixon S., Dantas J.A.R. (2017). Best practice for community-based management of postnatal depression in developing countries: a systematic review. Health Care Women Int.

[95] Yin J., Nisar A., Waqas A. (2020). Psychosocial interventions on perinatal depression in China: a systematic review and meta-analysis. J Affect Disord.

[96] Jidong D.E., Husain N., Roche A. (2021). Psychological interventions for maternal depression among women of African and Caribbean origin: a systematic review. BMC Wom Health.

[97] Huang R., Yan C., Tian Y. (2020). Effectiveness of peer support intervention on perinatal depression: a systematic review and meta-analysis. J Affect Disord.

[98] Shortis E., Warrington D., Whittaker P. (2020). The efficacy of cognitive behavioral therapy for the treatment of antenatal depression: a systematic review. J Affect Disord.

[99] McQueston K., Silverman R., Glassman A. (2013). The efficacy of interventions to reduce adolescent childbearing in low- and middle-income countries: a systematic review. Stud Fam Plann.

[100] Lassi Z.S., Kedzior S.G., Bhutta Z.A. (2019). Community-based maternal and newborn educational care packages for improving neonatal health and survival in low- and middle-income countries. Cochrane Database Syst Rev.

[101] Sarkar A., Chandra-Mouli V., Jain K., Behera J., Mishra S.K., Mehra S. (2015). Community based reproductive health interventions for young married couples in resource-constrained settings: a systematic review. BMC Public Health.

[102] Hindin M.J., Kalamar A.M., Thompson T.A., Upadhyay U.D. (2016). Interventions to prevent unintended and repeat pregnancy among young people in low- and middle-income countries: a systematic review of the published and gray literature. J Adolesc Health.

[103] Mason-Jones A.J., Sinclair D., Mathews C., Kagee A., Hillman A., Lombard C. (2016). School-based interventions for preventing HIV, sexually transmitted infections, and pregnancy in adolescents. Cochrane Database Syst Rev.

[104] Oringanje C., Meremikwu M.M., Eko H., Esu E., Meremikwu A., Ehiri J.E. (2016). Interventions for preventing unintended pregnancies among adolescents. Cochrane Database Syst Rev.

[105] Ahinkorah B.O., Kang M., Perry L., Brooks F. (2020). Prevention of adolescent pregnancy in anglophone sub-saharan Africa: a scoping review of national policies. Int J Health Policy Manag.

[106] Nkhoma D.E., Lin C.P., Katengeza H.L. (2020). Girls’ empowerment and adolescent pregnancy: a systematic review. Int J Environ Res Public Health.

[107] Mbuagbaw L., Medley N., Darzi A.J., Richardson M., Garga K.H., Ongolo-Zogo P. (2015). Health system and community level interventions for improving antenatal care coverage and health outcomes. Cochrane Database Syst Rev.

[108] Jacobs W., Downey L.E. (2022). Impact of conditional cash transfer programmes on antenatal care service uptake in low and middle-income countries: a systematic review. BMJ Open.

[109] Vanhuyse F., Stirrup O., Odhiambo A. (2022). Effectiveness of conditional cash transfers (Afya credits incentive) to retain women in the continuum of care during pregnancy, birth and the postnatal period in Kenya: a cluster-randomised trial. BMJ Open.

[110] United Nations United Nations sustainable development goals. The 17 goals. https://sdgs.un.org/goals.

[111] Brown K. (2011). ‘Vulnerability’: handle with care. Ethics Soc Welf.

[112] Clark B., Preto N. (2018). Exploring the concept of vulnerability in health care. CMAJ.

[113] Darroch J., Audam S., Biddlecom A. (2017). https://www.guttmacher.org/fact-sheet/adding-it-up-contraception-mnh-2017.

[114] Sully E., Biddlecom A., Darroch J. (2020). https://www.guttmacher.org/report/adding-it-up-investing-in-sexual-reproductive-health-2019.

[115] Grabovschi C., Loignon C., Fortin M. (2013). Mapping the concept of vulnerability related to health care disparities: a scoping review. BMC Health Serv Res.

