# Supplementary material for: Vulnerabilities and reparative strategies during pregnancy, childbirth, and the postpartum period: moving from rhetoric to action
Source: eClinicalMedicine. 2023 Dec 6;67:102264. doi: 10.1016/j.eclinm.2023.102264 (PMC10837549; doi:10.1016/j.eclinm.2023.102264)
Supplement: Appendices 1–5 and Supplementary Fig. S1 [file mmc1.pdf]

Supplemental Figure S1. Conceptual diagram demonstrating the interlinkages between vulnerability attributes of barriers (pale pink boxes), risk exposures (pink boxes), deficiency (green boxes) and their impact on maternal and perinatal outcomes (white boxes) during early pregnancy, late pregnancy and childbirth based on existing evidence.

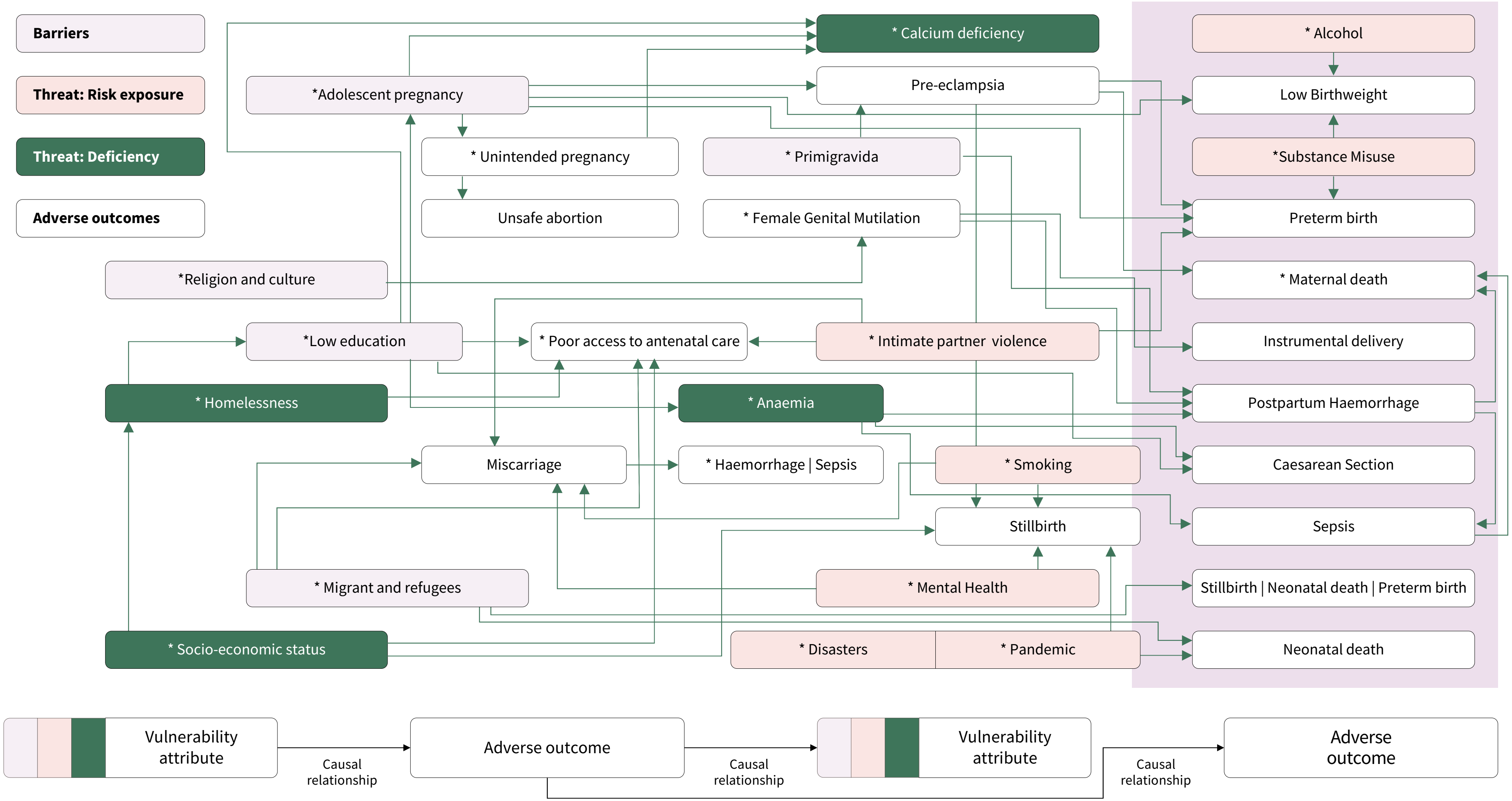

** Indicates variables where the relationships between threats and barriers and adverse outcomes are not restricted to any stage of pregnancy and apply throughout pregnancy. Adverse outcomes may be repeated and/or appear separately, where there are different pathways linking threats and barriers.*

Supplemental Table 1. Vulnerability attributes associated with adverse outcomes during pregnancy, childbirth, and postpartum periods in systematic reviews (see **Appendix 4** for full characteristics of included studies and references)

Vulnerability attribute	Early pregnancy	Late pregnancy	Childbirth	Other outcomes
**THREAT: DEFICIENCIES**				
**Anaemia**		Small for gestational age[1,2]<br>Low birthweight[3,4]<br>Preterm birth[3,4]<br>Pre-eclampsia[5,6]	Neonatal mortality[3]<br>Postpartum haemorrhage[7]<br>Infection after caesarean section[8]	
**Calcium deficiency**		Pre-eclampsia[9–11]		
**Socioeconomic status**		Stillbirth[12]<br>Preterm birth[13,14]<br>Low birthweight[13,15]		
**Homelessness**	Access to antenatal care[16,17]	Preterm birth[17]<br>Low birthweight[17]	Birth complications including pre-eclampsia, chorioamnionitis, placental abruption, hypertensive disorders of pregnancy, iron deficiency or other anaemia[17]	
**THREAT: RISK EXPOSURE**				
**Intimate partner violence**	Miscarriage[18]<br>Access to antenatal care[19]<br>Termination of pregnancy[20]	Pre-eclampsia[18]<br>Preterm birth[18,21,22]<br>Low birthweight[18,22,23]<br>Stillbirth[18]	Neonatal mortality[18]<br>Perinatal depression[24]	Postnatal depressive symptoms[25]
**Disasters**	Access to antenatal care (pandemic)[26]	Stillbirth (pandemic)[26]<br>Pre-eclampsia (terrorist attack)[27]<br>Low birthweight (terrorist attack;[27] natural disasters;[28] wildfires)[29,30]	Reduced institutional delivery (pandemic)[26]<br>Neonatal mortality (pandemic)[26]<br>Perinatal depression (earthquakes)[31,32]	Psychosis[33]
**Political instability/ Fragile and conflict-affected state (FCAS)**	Access to antenatal service[34,35]	Stillbirth[36]<br>Low birthweight[36]		Congenital birth defects[37]

**Substance misuse**		Low birthweight[38–42]<br>Preterm birth[42]		
**Alcohol**		Low birthweight[43–45]<br>Placenta abruption[46]		
**Smoking**	Miscarriage[47]	Stillbirth[48,49]	Neonatal mortality[49]	
**Mental health**		Low birthweight[50,51]<br>Preterm birth[50,51]		
**BARRIERS**				
**Adolescent pregnancy**	Anaemia[52]	Pre-eclampsia[53,54]<br>Preterm birth[52–55]<br>Low birthweight[52–55]	Fetal distress[52]<br>Obstructed labour[55]	
**Religion and culture**		Low birthweight (fasting mothers)[56]<br>Lower placental weight in fasting mothers[57]	Prolonged labour (Female Genital Mutilation (FGM))[58,59]<br>Instrumental delivery (FGM)[58]<br>Postpartum haemorrhage (FGM)[58]<br>Perineal tears (FGM)[59,60]<br>Fetal distress (FGM)[60]<br>Complications (peripartum hysterectomy; uterine rupture; prolonged labour) associated with faith-based institutions (e.g., spiritual churches)[61]	
**Low education**	Access to antenatal care[62,63]<br>Childhood pregnancies[64]<br>Unintended pregnancies[65,66]	Stillbirth[12]<br>Hypertensive disorders in pregnancy[5]<br>Anaemia[67]	Neonatal mortality[68]<br>Preparation for birth and emergencies[69]	Breastfeeding[70]
**Primigravida or grand multiparity**		Pre-eclampsia (primigravida)[5,6,71]<br>Gestational diabetes[72]	Postpartum haemorrhage[73–75]<br>Perinatal depression[76] Uterine rupture[77]	

**Migrants**	Access to antenatal care[78,79]<br>Miscarriage[78]	Pre-eclampsia[78]<br>Low birthweight[78]<br>Gestational diabetes[80]<br>Small for gestational age[78]<br>Stillbirth[78]<br>Preterm birth[79]	Neonatal mortality[78,79]<br>Maternal mortality[78]	Infection[79]<br>Caesarean section[78,79]
**Refugees**	Miscarriage[81]<br>Access to antenatal service[82]	Stillbirth[81–83]<br>Preterm birth[81,82]	Neonatal mortality[82,83]<br>Maternal death (hypertensive disorders, deep vein thrombosis and pre-eclampsia)[82]	Mental health conditions[82]
**Child marriage**			Reduced institutional delivery[84]	Intimate partner violence[84]

Appendices

Appendix 1. Vulnerability framework in pregnancy review: OVID search strategy for Medline, EMBASE and APA PsycInfo databases (searched 15 August 2022)

Appendix 2. Literature search terms used for identification of systematic reviews

Appendix 3. Flow chart of systematic reviews identified.

Appendix 4a-c. Association between different vulnerability attributes and adverse outcomes in early pregnancy (4a), late pregnancy (4b) and childbirth (4c).

Appendix 5. Reparative interventions to address various vulnerability attributes and improvement in pregnancy outcomes

Appendix 1. Vulnerability framework in pregnancy review: OVID search strategy for Medline, EMBASE and APA PsycInfo databases (searched 15 August 2022)

Embase <1974 to 2022 August 15>, Ovid MEDLINE(R) <1946 to August Week 3 2022>, APA PsycInfo <1967 to August Week 2 2022>

#	Query	Results
1	(vulnerability or vulnerab*).ti,ab.	455962
2	(systematic review or scoping review or literature review).ti,ab.	813440
3	(Pregnancy or Pregnan* or pregnant women or Antenat* or Birth or Childbirth or Postnat* or maternal health or maternal*).ti,ab.	2394417
4	1 AND 3	37456
5	concept analysis.ti,ab.	5090
6	2 AND 5	818020
7	4 AND 6	1016
8	Remove duplicates from 7	578

Appendix 2. Literature search terms used for identification of systematic reviews

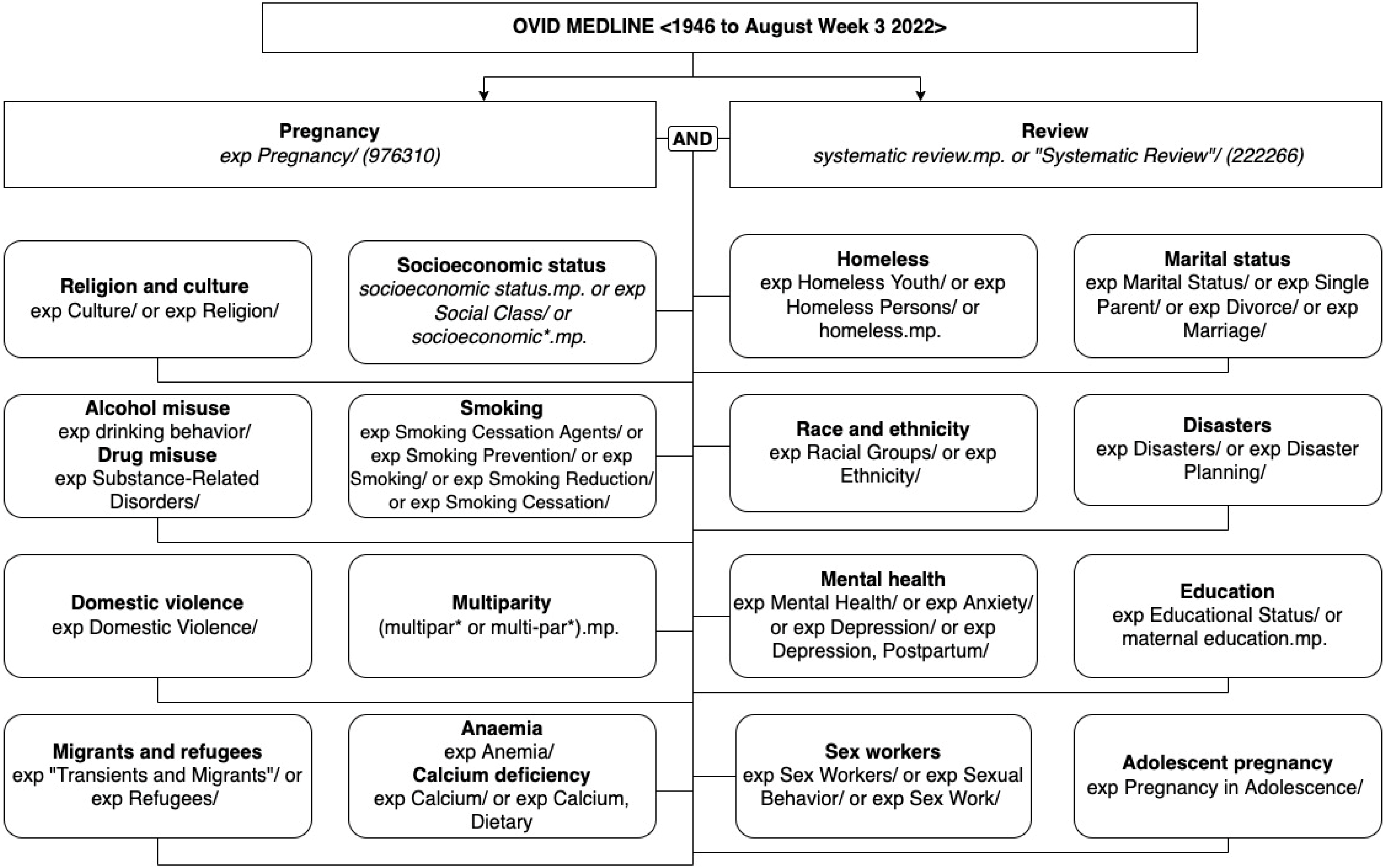

Appendix 3. Flow chart of systematic reviews identified.

BARRIERS

Vulnerability attributes	Adolescent pregnancy	Race and ethnicity	Religion and culture	Education	Multiparity	Migrants and refugees	Marital status
Number of citations identified	87	130	104	63	99	30	26
**Title and Abstract Screening**							
Number of citations retrieved	39	30	12	31	28	7	7
**Full-text Screening**							
Number of included reviews	13	0	6	17	10	6	0

THREAT: RISK EXPOSURE

Vulnerability attributes	Alcohol misuse	Drug misuse	Smoking	Disasters	Intimate partner violence	Sex workers	Mental health
Number of citations identified	89	164	194	30	57	122	93
**Title and Abstract Screening**							
Number of citations retrieved	8	18	26	10	18	1	15
**Full-text Screening**							
Number of included reviews	4	5	4	8	8	0	10

THREAT: DEFICIENCIES

Vulnerability attributes	Anaemia	Homelessness	Socio-economic status	Calcium deficiency
Number of citations identified	191	3	222	52
**Title and Abstract Screening**				
Number of citations retrieved	25	2	20	31
**Full-text Screening**				
Number of included reviews	16	2	4	3

Appendix 4a-c. Association between different vulnerability attributes and adverse outcomes in early pregnancy (4a), late pregnancy (4b) and childbirth (4c).

A. Early Pregnancy

Vulnerability attribute	Outcome	Study (Author Year)	Study Country or Region	Synthesis	Effect Estimates OR/RR (95% CI)
**BARRIERS**					
Adolescent pregnancy	Anaemia	Karacam 2021[52]	Turkey	Meta-analysis	2.60 (1.56-4.32) [10 studies]
Maternal Education	Access to antenatal care	Banke-Thomas 2017[62]	Bangladesh, Benin, Bolivia, Brazil, Burkina Faso, Cambodia, Cameroon, Chad, Comoros, Ethiopia, Ghana, Guatemala, Guinea, India, Indonesia, Ivory Coast, Kenya, Madagascar, Malawi, Mali, Mozambique, Nepal, Nicaragua, Niger, Nigeria, Peru, Senegal, Tanzania, Togo, Uganda, Zambia and Zimbabwe	Narrative	8/9 studies show significant differences (OR not documented)
		Tesfaye G 2017[63]	Ethiopia	Meta-analysis	0.49 (0.38-0.63) [18 studies]
	Adolescent pregnancy	Kassa GM 2018[64]	24 African countries (18 West Africa, 19 East Africa, 7 Central Africa and 6 South Africa) Specific countries not reported.	Meta-analysis	Not attending school: 2.49 (1.58-3.92) [10 studies]<br>No maternal education: 1.88 (1.29-2.73) [5 studies]
	Unintended pregnancy	Alene M 2020[65]	Ethiopia	Meta-analysis	10/21 studies significant difference (OR not documented)

		Bain LE 2020[66]	Senegal, Tanzania, Ghana, Kenya, Ethiopia, Nigeria, Democratic Republic of Congo, South Africa, Zambia and Malawi	Narrative	4/9 studies shown significant differences (OR not documented)
Multiparity	Gestational Diabetes Mellitus	Lee KW 2018[72]	India, Iran, China, Saudi Arabia, Thailand, Sri Lanka, Bangladesh, Israel, Vietnam, Malaysia, Qatar, Pakistan, Nepal, Yemen, Hong Kong, Singapore, Taiwan, Turkmenistan	Meta-analysis	Multiparity ≥2: 1.37 (1.24-1.52) [32 studies]
Migration and refugees	Access to antenatal care	Gagnon 2009[79]	Asian and Sub-Saharan migrants to receiving countries (USA, UK, France, Italy, Norway, Australia, Sweden, Spain, the Netherlands, Belgium, Germany, Portugal, Canada, Croatia, Greece, Ireland, Switzerland)	Narrative	Migrant group outcomes were 9.1% worse, 72.7% better, 18.2% mixed, and 0% had no difference [12 studies]
		Almeida LM 2013[78]	Netherlands (Africans, Surinam, Netherlands, Antilles/West Caribbean, Turkey, Morocco, Somalia, other Sub-Saharan countries, Indonesia, Vietnam migrants)	Narrative	Effect estimate OR 1.65 (0.96 to 2.82) to RR 7.8 (0.6-106.2) [2 studies]
		Heslehurst N 2018[82]	Not documented.	Narrative	16/16 studies showed a positive association between migrant women and inadequate prenatal care compared to host country women. 15/29 studies in one review had aOR >2.0 [1 study]

					Structural and organisational barriers: 10/16 studies<br>Social barriers: 9/16 studies<br>Personal and cultural barriers: 6/16 studies
	Miscarriage	Almeida LM 2013[78]	Italy (Eastern Europe, Africa, Asia, South America migrants)	Narrative	Risk ratio: 0.15 for 'regular migrants' vs 0.35 for 'irregular migrants' vs 0.16 for host country [1 study]
		Harakow HI 2021[81]	Germany, Pakistan	Narrative	OR 1.56 to 1.58 [2 studies]
**THREAT: RISK EXPOSURE**					
Intimate partner violence	Miscarriage	Han A 2013[18]	Brazil	Narrative	OR 1.88 (1.25-2.82) [1 study]
	Access to antenatal care	Han A 2013[18]	Brazil, Mexico	Narrative	OR 2.20 (1.10-4.40) [2 studies]
	Termination of pregnancy (TOP)	Hall M 2014[20]	Not documented (LMICs)	Meta-analysis	IPV and partner without knowledge of TOP: OR 2.32 (2.00-2.69) [2 studies]<br>IPV and partner support for TOP: OR 1.37 (0.82-2.30) [3 studies]
Disasters (pandemic)	Access to antenatal care	Palo SK 2022[26]	India, Liberia, Ethiopia, China	Narrative	Fewer antenatal care appointments attended during the COVID-19 pandemic [4 studies]
Political instability/ Fragile	Access to antenatal service	Gopalan SS 2017[35]	Nepal, Yemen	Narrative	Maintained antenatal care usage [3 studies]; At least one ANC visit

and conflict-affected states (FCAS)					was above 50% in Nepal and Yemen.
		Alibhai KM 2022[34]	Fragile and conflict-affected states	Narrative	Poor quality of ANC: 49/121 studies<br>Distance: 47/121 studies<br>Transportation: 14/121 studies<br>Infrastructure/resources: 11/121 studies
Smoking	Miscarriage	Pineles BL 2014[47]	Brazil	Narrative	OR 0.66 to 1.33 [2/3 studies]
**THREAT: DEFICIENCIES**					
Homelessness	Access to antenatal care	McGeough C 2020[16]	Iran, Turkey	Narrative	Barriers to accessing health system (OR not reported) [2/2 studies]
		Di Tosto 2021[17]	USA	Narrative	First-trimester prenatal visit: aOR 2.00 (1.90-2.20) [1 study]<br>Late prenatal care: aOR 1.75 (1.63-1.90) [2 studies]

OR, odds ratios; RR, relative risk; aOR, adjusted odds ratios; 95% CI, 95% confidence intervals

B. Late Pregnancy

Vulnerability attribute	Outcome	Study (Author Year)	Region	Synthesis	Effect Estimates OR/RR (95% CI)
**BARRIERS**					
Adolescent pregnancy	Pre-eclampsia	de Azevedo WF 2015[54]	Brazil, Cameroon, England, Germany, Iran, Nepal, Tanzania, USA	Narrative	OR 1.82 to 4.38 [2 studies]
		Grønvik T 2018[53]	Cameroon, Nigeria, Tanzania, South Africa, Zambia, Namibia, Gabon, Ghana, Ethiopia, Sudan.	Meta-analysis	OR 3.52 (2.26-5.48) [3 studies]
	Preterm birth	de Azevedo WF 2015[54]	Brazil, Cameroon, England, Germany, Iran, Nepal, Tanzania, USA	Narrative	Prevalence 3.7 to 39.4% (OR not reported) [10 studies]
		Williamson N 2013[55]	Not specified (LMICs)	Narrative	OR not reported
		Grønvik T 2018[53]	Cameroon, Nigeria, Tanzania, South Africa, Zambia, Namibia, Gabon, Ghana, Ethiopia, Sudan.	Meta-analysis	OR 1.75 (1.18-2.61) [5 studies]
		Karacam 2021[52]	Turkey	Meta-analysis	OR 2.12 (1.64-2.72) [29 studies]
	Low birthweight	de Azevedo WF 2015[54]	Brazil, Cameroon, England, Germany, Iran, Nepal, Tanzania, USA	Narrative	OR 0.96 to 2.70 [9 studies]
		Williamson N 2013[55]	Not specified (LMICs)	Narrative	OR not reported
		Grønvik T 2018[53]	Cameroon, Nigeria, Tanzania, South Africa,	Meta-analysis	OR 1.61 (1.24-2.09) [10 studies]

			Zambia, Namibia, Gabon, Ghana, Ethiopia, Sudan.		
		Karacam 2021[52]	Turkey	Meta-analysis	2.06 (1.59-2.68) [25 studies]
Religion and culture	Low birthweight (fasting mothers)	Oosterwijk VNL 2021[56]	Burkina Faso, Canada, Egypt, England, Indonesia, Iran, Iraq, Lebanon, Netherlands, Saudi Arabia, Tunisia, Turkey, UAE, Uganda,	Narrative	LBW in fasting mothers (OR not reported) [1/14 studies]
Education	Stillbirth	Di Mario S 2007[12]	Not specified (LMICs)	Narrative	OR 2.30 to 8.50 [2 studies]
	Hypertensive disorders in pregnancy	Meazaw MW 2020a[6]	Nigeria, Ethiopia, South Africa, Sudan (Sub-Saharan Africa)	Meta-analysis	OR 1.12 (0.59–1.65) [7 studies]
	Anaemia	Ali SA 2020[67]	Not specified (South Asia and Africa)	Narrative	Secondary education: OR 0.769 (0.919-0.644); Higher education: OR 0.638 (0.870-0.468) [1/5 studies]
Primigravida	Pre-eclampsia (primigravida)	Luo ZC 2007[71]	Australia, Denmark, Finland, Norway, Saudi Arabia, Taiwan, UK, USA, Zimbabwe	Meta-analysis	OR 2.42 (2.16 – 2.71) [23 studies]
		Meazaw MW 2020a[6]	Nigeria, Ethiopia, South Africa, Sudan (Sub-Saharan Africa)	Meta-analysis	OR 2.52 (1.19 – 3.86) [6 studies]

	(Hypertensive disorders in pregnancy)	Meazaw MW 2020[5]	Ethiopia, Nigeria and Ghana (Sub-Saharan Africa)	Meta-analysis	OR 1.78 (1.11 – 2.44) [4 studies]
	Gestational diabetes	Lee KW 2018[72]	India, Iran, China, Saudi Arabia, Thailand, Sri Lanka, Bangladesh, Israel, Vietnam, Malaysia, Qatar, Pakistan, Nepal, Yemen, Hong Kong, Singapore, Taiwan, Turkmenistan	Meta-analysis	OR 0.55 (0.41-0.73) [18 studies]
Migration	Pre-eclampsia	Almeida LM 2013[78]	Germany (Middle and Northern Europe/North America, Middle East/North Africa, Asia, and Eastern Europe migrants)	Narrative	Higher rates of pre-eclampsia in German (host country) women (2.5%) than in migrant populations of Middle East and North African women (1.3%) and Asian women (1.2%) [1 study].
			Netherlands (Western, Non-western, Surinam/Dutch Antilles, Turkey, Morocco, Other (Sub-Saharan Africa, Asia) migrants)	Narrative	Lower rates of pre-eclampsia as a cause of death in migrant women vs host country (87 vs 94%) [1 study]
	Low birthweight (LBW)	Almeida LM 2013[78]	Spain (Central and South America, Magreb,	Narrative	Lower rates of LBW in migrants (3.8% vs 10%, OR 0.17 (0.03-0.90)) [1 study]

		Caribbean-Africa, Southeast Asia/ Filipinas, India/Pakistan, Eastern Europe, China migrants)		
Gestational diabetes	Gagnon AJ 2011[80]	Receiving countries: US, UK, UAE, Israel, Norway, Australia, Spain, Bahrain, The Netherlands, Austria	Meta-analysis	Western Asian: OR 1.79 (1.27-2.51) [7 studies] African migrants: OR 2.46 (2.12-2.85) [4 studies]
Small for gestational age	Almeida LM 2013[78]	Finland	Narrative	Higher rates in migrants (2.7% vs 2.0%)
Stillbirth	Almeida LM 2013[78]	Germany (Middle and Northern Europe/North America, Middle East/North Africa, Asia, Eastern Europe migrants)	Narrative	Higher rate of stillbirth: Middle East and North African women, RR 1.34 (1.22-1.55); Asian women, RR 1.34 (1.02-1.65); Mediterranean women, RR 1.14 (0.93-1.28)
Preterm birth	Gagnon AJ 2009[79]	Asian and Sub-Saharan migrants to receiving countries (USA, UK, France, Italy, Norway, Australia, Sweden, Spain, the Netherlands, Belgium, Germany, Portugal, Canada, Croatia, Greece, Ireland, Switzerland)	Meta-analysis	Any ‘immigrant’ vs ‘majority’ receiving country population: OR 1.09 (0.87–1.36) [4 studies]

Refugees	Stillbirth	Gissler M 2009[83]	Host countries and regions: Australia, Europe, USA	Meta-analysis	Migrant women vs host country women: RR 1.40 (1.22-1.58) [6 studies] Non-European migrants vs European migrants: RR 1.88, 1.58-2.23) [5 studies]
		Harakow HI 2021[81]	Australia, Belgium, Canada, Finland, Germany, Israel, Jordan, Norway, Pakistan, Sweden, Turkey, USA	Meta-analysis	RR 1.20 to 2.24 [6/10 studies]
		Heslehurst N 2018[82]	Host countries and regions: Australia, Europe, USA	Narrative	Migrant women vs host country women: RR 1.40 (1.22-1.58) [1 study: Gissley M 2009] Non-European migrants vs European migrants: RR 1.88, 1.58-2.23) [1 study: Gissler M 2009]
	Preterm birth	Harakow HI 2021[81]	Not documented	Narrative	RR 0.61 to 0.92 [9/10 studies]
		Heslehurst N 2018[82]	Not documented	Narrative	OR 0.83 to 1.29 [3 studies]
**THREAT: RISK EXPOSURE**					
Intimate partner violence	Pre-eclampsia	Han A 2013[18]	Bolivia, Brazil, Columbia, Costa Rica, Dominican Republic, Guatemala, Haiti, Mexico, Nicaragua	Narrative	OR 2.7 (1.9 – 3.9) [1 study]

	Preterm birth	Han A 2013[18]	Bolivia, Brazil, Columbia, Costa Rica, Dominican Republic, Guatemala, Haiti, Mexico, Nicaragua	Narrative	OR not reported [0/7 studies]
		Parys ASV 2014[21]	Australia, Hawaii, Hong Kong, Peru, USA	Narrative	Fewer preterm neonates (1.5% vs 6.6%, p=0.03) [1 study]
		Kern A 2022[22]	Brazil	Narrative	OR 1.21 to 1.36 [2 studies]
	Low birthweight	Han A 2013[18]	Bolivia, Brazil, Columbia, Costa Rica, Dominican Republic, Guatemala, Haiti, Mexico, Nicaragua	Narrative	OR 3.3 to 4.0 [3/5 studies]
		Hill A 2016[23]	Nicaragua	Narrative	OR 3.98 (1.70-9.31) [1 study]
		Kern A 2022[22]	Brazil	Narrative	OR 1.43 to 2.18 [22 studies]
	Stillbirth	Han A 2013[18]	Haiti	Narrative	Spousal violence 40.8% vs never experienced spousal violence 24.8% [1 study]
Disasters	Stillbirth (pandemic)	Palo SK 2022[26]	India, Nepal, Guinea	Narrative	Rise in stillbirth rate (No OR reported) [3 studies]
	Pre-eclampsia (terrorist attack)	Harville EW 2010[27]	Colombia, Serbia (Netherlands, Spain, USA)	Narrative	aOR 0.60 (0.41 – 0.86) [1 study]
	Low birthweight (terrorist attack)	Harville EW 2010[27]	Colombia, Serbia (Netherlands, Spain, USA)	Narrative	aOR 0.83 to 1.34 [1/5 studies]

	Low birthweight (natural disasters)	Sanguanklin N 2014[28]	Thailand	Primary study	Variance in infant birthweight, $F(6, 168) = 3.24$ (p=0.005)
	Low birthweight (wildfires)	Jayachandran S 2009[30]	Indonesia	Primary study	Air pollution linked to fetal growth retardation or shorter gestation period which are associated with LBW (No OR reported) [3 studies]
		Amjad S 2021[29]	Australia, Brazil, Indonesia, USA	Narrative	No OR reported [6/7 studies]
Political instability/ Fragile and conflict-affected state (FCAS)	Stillbirth	Keasley J 2017[36]	Iraq, Kuwait, Libya, Bosnia and Herzegovina, Israel, Palestine, Kosovo and Yugoslavia, Nepal, Somalia, Afghanistan	Narrative	No OR reported [1/5 studies]
	Low birthweight	Keasley J 2017[36]	Iraq, Kuwait, Libya, Bosnia and Herzegovina, Israel, Palestine, Kosovo and Yugoslavia, Nepal, Somalia, Afghanistan	Narrative	OR 3.00 to 3.97 [9/9 studies]
Substance misuse	Low birthweight	Hayes JS 1988[41]	Jamaica	Primary study	Birthweight in non-users: 3074.08 (SD 528.98) Irregular marijuana users: 3151.97 (SD 429.31) Moderate users: 3020.50 (SD 439.22) Heavy users: 3277.91 (SD 384.21)

					(No effect size reported)
		Chomchai C 2004[39]	Thailand	Primary study	Regression coefficient = -217.9 (-415.1, -20.7), p<0.05
		Ladhani NNN 2011[38]	Australia, Thailand, USA	Meta-analysis	OR 3.97 (2.45– 6.43) [2 studies]
		Conner SN 2016[40]	Australia, Canada, Jamaica, Netherlands, New Zealand, Spain, UK, USA	Meta-analysis	RR 1.43 (1.27 – 1.62) [12 studies]
		Marchand G 2022[42]	Canada, Jamaica, USA	Meta-analysis	RR 2.06 (1.25-3.42) [7 studies]
	Preterm birth	Marchand G 2022[42]	Canada, Jamaica, USA	Meta-analysis	RR 1.28 (1.16-1.42) [12 studies]
Alcohol	Low birthweight	Jackson DJ 2007[45]	South Africa	Primary study	aOR 1.32 (0.80 – 2.20)
		da Silva I 2011[44]	Brazil	Primary study	OR 4.20 (1.25 -14.13)
		Pereira PPS 2019[43]	Africa, Americas, Asia, Europe, Oceania	Narrative	OR 1.62 to 4.20 [3/5 studies]
	Placenta Abruption	Steane SE 2021[46]	Tanzania	Meta-analysis	aOR 1.48 (1.37-1.60) [4 study]
Smoking	Stillbirth	Marufu TC 2015[48]	Brazil, Canada, Denmark, Finland, India, New Zealand, Sweden, UK, USA	Meta-analysis	OR 1.47 (1.37 – 1.57) [25 studies]
		Pineles BL 2016[49]	Not documented	Meta-analysis	RR 1.46 (1.38-1.54) [42 studies]

Mental Health	Low birthweight	Dadi AF 2020[50]	LMICs (Pakistan, Bangladesh, Vietnam, Peru, Korea, India, Ghana, Ethiopia)	Meta-analysis	RR 1.66 (1.06-2.61) [6 studies]
		Dadi AF (1) 2020[51]	LMICs (Not specified)	Meta-analysis	Pooled OR 1.39 (1.11-1.58) [3 studies]
	Preterm birth	Dadi AF 2020[50]	LMICs (Pakistan, Bangladesh, Vietnam, Peru, Korea, India, Ghana, Ethiopia)	Meta-analysis	RR 2.68 (1.89-2.92) [4 studies]
		Dadi AF (1) 2020[51]	LMICs (Not specified)	Meta-analysis	Pooled OR 1.49 (1.32-1.68) [3 studies]
**THREAT: DEFICIENCIES**					
Homelessness	Preterm birth	Di Tosto 2021[17]	USA	Narrative	aOR 0.73 to 1.43 [4 studies]
	Low birthweight	Di Tosto 2021[17]	USA	Narrative	No OR reported [10 studies]
Anaemia	Small for gestational age	Kozuki N 2012[1]	China, Finland, Korea, Malawi, Peru, Sri Lanka, UK, USA	Meta-analysis	Moderate to severe anaemia (<90 or <80-g/L): OR 1.53 (1.24–1.87) [5 studies]
		Badfar G 2019[2]	China, Finland, India, Peru	Meta-analysis	RR 1.11 (0.99 – 1.24) [7 studies]
	Low birthweight	Rahman MM 2016[3]	East-West Asia, South Asia, Africa, and South America	Meta-analysis	RR 1.31 (1.13 -1.51) [17 studies]

		Jung J 2019[4]	Africa, Eastern Mediterranean, Europe, Southeast Asia, Western Pacific	Meta-analysis	OR 1.65 (1.45 –1.87) [56 studies]
	Preterm birth	Rahman MM 2016[3]	East-West Asia, South Asia, Africa, and South America	Meta-analysis	RR 1.63 (1.33-2.01) [13 studies]
		Jung J 2019[4]	Africa, Eastern Mediterranean, Europe, Southeast Asia, Western Pacific	Meta-analysis	OR 2.11 (1.76 – 2.53) [50 studies]
	Pre-eclampsia	Meazaw MW 2020a[6]	Nigeria, Ethiopia, South Africa, Sudan (Sub-Saharan Africa)	Meta-analysis	OR 3.22 (2.70 - 3.75) [5 studies]
Calcium deficiency	Pre-eclampsia	Segovia BL 2004[9]	Brazil	Primary study	RR 4.06 (3.29-7.20)
		Schoenaker DAJM 2014[11]	Not documented	Meta-analysis	OR 0.76 (0.57-1.01) [7 studies]
		Hofmeyr GJ 2018[10]	Philippines, Iran, Trinidad, Argentina, China, Australia, Colombia, India, Ecuador, Hong Kong, Indonesia, USA	Narrative	No OR reported [27 studies]
Socioeconomic status	Stillbirth	Di Mario S 2007[12]	Not specified (LMICs)	Narrative	OR 1.8 to 16.2 [3/5 studies]
	Preterm birth	Muhihi A 2016[14]	Tanzania	Primary study	Wealth quintile (Q5 reference): Q1, RR 1.35

					(1.12-1.64); Q2 RR 1.21 (1.01-1.45)
		Ngandu CB 2020[13]	Sub-Saharan Africa	Narrative	Maternal occupation: OR 0.43 to 2.95 [1 study]<br><br>Household wealth index: aRR 1.08 to 1.49 [1 study]<br><br>Maternal education: aRR 0.95 to 1.74 [2 studies]
	Low birthweight	Assefa N 2012[15]	Ethiopia	Primary study	OR 2.1 (1.42 - 3.05)
		Ngandu *C*B 2020[13]	Sub-Saharan Africa	Narrative	Maternal education: aOR 0.9 to 1.2 [1 study]<br><br>Household wealth index: aOR 1.0 to 2.1 [1 study]<br><br>Maternal occupation: aOR 0.28 to 0.45 [1 study]

OR, odds ratios; RR, relative risk; aOR, adjusted odds ratios; 95% CI, 95% confidence intervals

C. Childbirth and postpartum

Vulnerability attribute	Outcome	Study (Author Year)	Region	Synthesis	Effect Estimates OR/RR (95% CI)
**BARRIERS**					
Adolescent pregnancy	Fetal distress	Karacam Z 2021[52]	Turkey	Meta-analysis	OR 1.78 (1.22-2.59) [14 studies]
	Obstructed labour	Williamson N 2013[55]	Not specified (LMICs)	Narrative	No OR was reported.
Religion and culture	Prolonged labour (FGM)	Berg RC 2014[58]	Kenya, Nigeria, Norway, Sweden	Meta-analysis	OR 1.49 (1.01-2.19) [4 studies]
		Lurie JM 2020[59]	Not specified (Africa, Asia)	Meta-analysis	OR 2.04 (1.27-3.28) [12 studies]
	Instrumental delivery (FGM)	Berg RC 2014[58]	USA, Norway	Meta-analysis	Primiparous women: OR 1.56 (1.32-1.86) [2 studies]<br>Multiparous women: OR 1.34 (0.80-2.26) [2 studies]
		Lurie JM 2020[59]	Africa, Asia	Meta-analysis	OR 1.18 (0.78-1.79) [14 studies]
	Postpartum haemorrhage (FGM)	Berg RC 2014[58]	Not specified	Meta-analysis	OR 1.50 (1.22-1.84) [5 studies]
	Caesarean section (FGM)	Berg RC 2014[58]	Not specified	Meta-analysis	OR 1.32 (0.97-1.80) [5 studies]
		Lurie JM 2020[59]	Africa, Asia	Meta-analysis	OR 1.51 (0.99-2.30) [25 studies]
	Difficult delivery (FGM)	Berg RC 2014[58]	Nigeria	Meta-analysis	OR 1.88 (1.06-3.35) [2 studies]
	Perineal tears (FGM)	Obermeyer CM 2005[60]	Not specified	Meta-analysis	OR 1.43 (95% CI not reported) [1 study]
		Berg RC 2014[58]	USA, Nigeria, Norway	Meta-analysis	OR 1.39 (0.99-1.95) [4 studies]
		Lurie JM 2020[59]	Europe, Africa	Meta-analysis	OR 2.63 (1.35-5.11) [8 studies]

	Fetal distress	Obermeyer CM 2005[60]	Not specified	Meta-analysis	OR 2.60 (95% CI not reported) [1 study]
	Peripartum complications (faith-based institutions)	Hirose A 2017[61]	Nigeria	Narrative	No OR reported [3 studies]
Education	Neonatal mortality	Ramaiya A 2014[68]	Kenya	Meta-analysis	OR 4.9 (3.14-7.68) [1 study]
	Preparation for birth and emergencies	Ketema DB 2020[69]	Ethiopia	Meta-analysis	OR 2.40 (1.9-3.1) [15 studies]
	Breastfeeding	Zhao J 2017[70]	China	Meta-analysis	Lower in mothers educated more than six years: OR 0.90 (0.83-0.97) [15 studies]
Primigravida or grand multiparity	Postpartum haemorrhage	Temesgen MA 2017[75]	Ethiopia	Primary study	Multiparity: aOR 4.3 (0.6-36.0)<br>Grand multiparity: aOR 12.4 (1.6-97.2)
		Habitamu D 2019[74]	Ethiopia	Primary study	Chi-square test on parity (1, 2-4 and >=5): 29.231(2), p<0.05
		Nigussie J 2022[73]	Ethiopia	Meta-analysis	Grand multiparity: OR 6.58 (1.90-22.80) [2 studies]
	Perinatal depression	Yang K 2022[76]	Not specified (LMIC)	Meta-analysis	OR 0.78 (0.63-0.96)
	Uterine rupture	Desta M 2020[77]	Ethiopia	Meta-analysis	Grand multiparity: OR 4.49 (2.83-7.11) [7 studies]
Migrants	Neonatal mortality	Gagnon A 2009[79]	Asian and Sub-Saharan migrants to receiving countries (USA, UK, France, Italy, Norway, Australia, Sweden, Spain, the Netherlands, Belgium, Germany,	Meta-analysis	Any 'immigrant' vs 'majority' receiving country population: OR 1.07 (0.58-1.97) [2 studies]

		Portugal, Canada, Croatia, Greece, Ireland, Switzerland)		
	Almeida LM 2013[78]	Sweden (Sub-Saharan migrants)	Narrative	Perinatal death from suboptimal factors: OR 18 (3.3-100) [1 study]
Caesarean section	Gagnon A 2009[79]	Asian and Sub-Saharan migrants to receiving countries (USA, UK, France, Italy, Norway, Australia, Sweden, Spain, the Netherlands, Belgium, Germany, Portugal, Canada, Croatia, Greece, Ireland, Switzerland)	Narrative	Migrant group outcome: 40.0% worse, 20.0% better, 12.0% mixed, 28.0% had no difference [25 studies]
	Almeida LM 2013[78]	Scotland (Czeck, Estonia, Hungary, Latvia, Lithuania, Poland, Slovakia, and Slovenia migrants); Finland (Nordic, Western, Eastern European, Baltic, North African, S. Asian, Chinese, Somali, Vietnamese, African, and Latin American migrants)	Narrative	Lower elective caesarean section rate in migrant population (6 vs 10%) [1 study]<br>Less caesarean section deliveries in migrant population (18.2 vs 19.7%) [1 study]

	Maternal mortality	Almeida LM 2013[78]	France (Sub-Saharan African, Asian, North and South American migrants)	Narrative	OR 2.00 (1.42-2.80) [1 study]
			Netherlands (Non-western, Surinam/Dutch Antilles, Turkish, Moroccan, Other (Sub-Saharan African, Asian) migrants)	Narrative	Non-western women: OR 2.1 (1.6-2.7); Surinam-D.Antillean women: OR 2.7 (1.7-4.3); Other migrant women populations: OR 3.3 (2.3-4.8) [1 study]
Refugees	Neonatal mortality	Gissler M 2009[83]	Europe	Meta-analysis	RR 2.77 (1.47-2.73) [4 studies]
		Heslehurst N 2018[82]	Not documented	Narrative	RR 1.25 to 1.40 [3 studies]
	Maternal mortality	Heslehurst N 2018[82]	Not documented	Narrative	RR 2.00 (1.72-2.33) and absolute risk difference 9/100,000 deliveries per year for migrant women (5.9-15.2) [2 studies]
Child marriage	Institutional delivery	Fan S 2022[84]	South Asia, Sub-Saharan Africa	Narrative	Reduced likelihood of delivery in healthcare facility (No OR reported) [9 studies]
**THREAT: RISK EXPOSURE**					
Intimate partner violence (IPV)	Neonatal mortality	Han A 2013[18]	Brazil	Narrative	IPV 9.1% vs no IPV 2.2%, p=0.006 [1 study]
	Perinatal depression	Alvarez-Segura M 2014[24]	India, Pakistan, Thailand	Narrative	Increased (No OR reported) [4 studies]
Disasters	Institutional delivery (pandemic)	Palo SK 2022[26]	Guinea, India, Nepal	Narrative	Reduced (No OR reported) [9/13 studies]
	Neonatal mortality rate (pandemic)	Palo SK 2022[26]	Nepal, South Africa	Narrative	Increase in neonatal mortality (OR not reported) [3/3 studies]
	Perinatal depression (earthquakes)	Ren JH 2014[31]	Not specified	Narrative	Increased (No OR reported) [8 studies]
		Khatri GK 2019[32]	China, Taiwan	Narrative	Psychological stress: 6 to 40.8% [7 studies]
Smoking	Neonatal mortality	Pineles 2016[49]	Not documented	Meta-analysis	RR 1.22 (1.14-1.30) [18 studies]

**THREAT: DEFICIENCIES**					
Homelessness	Pre-eclampsia	Di Tosto 2021[17]	USA	Narrative	OR 1.0 (0.7-1.3) [0/1 studies]
	Placental abruption	Di Tosto 2021[17]	USA	Narrative	OR 1.2 (0.8-1.7) [0/1 studies]
	Chorioamnionitis	Di Tosto 2021[17]	USA	Narrative	OR 0.9 (0.6-1.3) [0/1 studies]
Anaemia	Neonatal mortality	Rahman MM 2016[3]	Not specified (LMICs)	Meta-analysis	RR 2.72 (1.19-6.25) [2 studies]
	Postpartum haemorrhage	Omotayo MO 2021[7]	USA, Peru, Ethiopia, India, Nigeria, Egypt, Uganda	Meta-analysis	OR 1.39 (0.64-3.01) [8 studies]
	Postpartum haemorrhage (severe anaemia)	Omotayo MO 2021[7]	Ghana, Peru, UK, India, Egypt	Meta-analysis	OR 3.54 (1.20-10.4) [5 studies]
	Infection after caesarean section	Getaneh T 2020[8]	Ethiopia	Meta-analysis	OR 4.56 (2.88-7.22) [4 studies]

OR, odds ratios; RR, relative risk; aOR, adjusted odds ratios; 95% CI, 95% confidence intervals

Appendix 5. Reparative interventions to address various vulnerability attributes and improvement in pregnancy outcomes

Vulnerability attribute	Intervention	Improved pregnancy outcome
**THREAT: DEFICIENCIES**		
**Anaemia**	Iron supplementation	Anaemia at term[85]
		Iron deficiency[85–89]
		Low birthweight[90,91]
		Maternal death[92]
		Neonatal mortality[92]
**Calcium deficiency**	Calcium supplementation	Pre-eclampsia[10]
		Pre-eclampsia-related morbidity and mortality[10]
**THREAT: RISK EXPOSURE**		
**Intimate partner violence**	Tailored intervention to improve independence, control, or safety planning.	Postnatal depression[93]<br>Safety behaviours[21]
**Smoking**	Psychosocial support (counselling)	Smoking cessation[94]
**Mental health**	Psychological interventions	Perinatal depression[95–100]
	Peer support intervention	Perinatal depression[101]
	Cognitive behaviour therapy	Perinatal depression[102]
**BARRIERS**		
**Low education**	Maternal education	Use of maternal health services[103–106]
		Neonatal mortality[107]
	Community health educational interventions (one-to-one or group counselling)	Perinatal mortality[107]<br>Initiation of breastfeeding[108]
**Unintended pregnancies**	Combined educational and contraceptive-promoting interventions	Unintended pregnancy among adolescents[109]

**Antenatal care access**	Community interventions (media campaigns, education and financial incentives)<br>Conditional cash transfers	Antenatal care attendance[110–112]

References

1. Kozuki N, Lee AC, Katz J, Child Health Epidemiology Reference Group. Moderate to severe, but not mild, maternal anemia is associated with increased risk of small-for-gestational-age outcomes. J Nutr. 2012;142(2):358–62.

2. Badfar G, Shohani M, Soleymani A, Azami M. Maternal anemia during pregnancy and small for gestational age: a systematic review and meta-analysis. J Matern Fetal Neonatal Med. 2019;32(10):1728–34.

3. Rahman MM, Abe SK, Rahman MS, Kanda M, Narita S, Bilano V, et al. Maternal anemia and risk of adverse birth and health outcomes in low- and middle-income countries: systematic review and meta-analysis. Am J Clin Nutr. 2016;103(2):495–504.

4. Jung J, Rahman MM, Rahman MS, et al. Effects of hemoglobin levels during pregnancy on adverse maternal and infant outcomes: a systematic review and meta-analysis. Ann N Y Acad Sci. 2019;1450(1):69-82.

5. Meazaw MW, Chojenta C, Muluneh MD, Loxton D. Factors associated with hypertensive disorders of pregnancy in sub-Saharan Africa: a systematic and meta-analysis. PLoS One. 2020;15(8):e0237476.

6. Meazaw MW, Chojenta C, Muluneh MD, Loxton D. Systematic and meta-analysis of factors associated with preeclampsia and eclampsia in sub-Saharan Africa. PLoS OnE. 2020 Aug 19;15(8):e0237600.

7. Omotayo MO, Abioye AI, Kuyebi M, Eke AC. Prenatal anemia and postpartum hemorrhage risk: A systematic review and meta-analysis. J Obstet Gynaecol Res. 2021;47(8):2565–76.

8. Getaneh T, Negesse A, Dessie G. Prevalence of surgical site infection and its associated factors after cesarean section in Ethiopia: systematic review and meta-analysis. BMC Pregnancy Childbirth. 2020;20(1):311.

9. Segovia BL, Vega IT, Villarreal EC, Licona NA. [Hypocalciuria during pregnancy as a risk factor of preeclampsia]. Ginecol Obstet Mex. 2004;72:570–4.

10. Hofmeyr GJ, Lawrie TA, Atallah ÁN, Torloni MR. Calcium supplementation during pregnancy for preventing hypertensive disorders and related problems. Cochrane Database Syst Rev. 2018;10(10):CD001059.

11. Schoenaker DA, Soedamah-Muthu SS, Mishra GD. The association between dietary factors and gestational hypertension and pre-eclampsia: a systematic review and meta-analysis of observational studies. BMC Med. 2014;12(1):157.

12. Di Mario S, Say L, Lincetto O. Risk factors for stillbirth in developing countries: a systematic review of the literature. Sex Transm Dis. 2007;34(7):S11–21.

13. Ngandu CB, Momberg D, Magan A, Chola L, Norris SA, Said-Mohamed R. The association between household socio-economic status, maternal socio-demographic characteristics and adverse birth and infant growth outcomes in sub-Saharan Africa: a systematic review. J Dev Orig Health Dis. 2020;11(4):317–34.

14. Muhihi A, Sudfeld CR, Smith ER, Noor RA, Mshamu S, Briegleb C, et al. Risk factors for small-for-gestational-age and preterm births among 19,269 Tanzanian newborns. BMC Pregnancy Childbirth. 2016;16(1):110.

15. Assefa N, Berhane Y, Worku A. Wealth status, mid upper arm circumference (MUAC) and antenatal care (ANC) are determinants for low birth weight in Kersa, Ethiopia. PLoS One. 2012;7(6):e39957.

16. McGeough C, Walsh A, Clyne B. Barriers and facilitators perceived by women while homeless and pregnant in accessing antenatal and or postnatal healthcare: a qualitative evidence synthesis. Health Soc Care Community. 2020;28(5):1380–93.

17. DiTosto JD, Holder K, Soyemi E, Beestrum M, Yee LM. Housing instability and adverse perinatal outcomes: a systematic review. Am J Obstet Gynecol. 2021;3(6):100477.

18. Han A, Stewart DE. Maternal and fetal outcomes of intimate partner violence associated with pregnancy in the Latin American and Caribbean region. Int J Gynecol Obstet. 2013;124(1):6–11.

19. Moraes CL, Arana FDN, Reichenheim ME. Violência física entre parceiros íntimos na gestação como fator de risco para a má qualidade do pré-natal. Rev Saúde Pública. 2010;44(4):667–76.

20. Hall M, Chappell LC, Parnell BL, Seed PT, Bewley S. Associations between intimate partner violence and termination of pregnancy: a systematic review and meta-analysis. PLoS Med. 2014;11(1):e1001581.

21. Parys ASV, Verhamme A, Temmerman M, Verstraelen H. Intimate partner violence and pregnancy: a systematic review of interventions. PLoS One. 2014;9(1):e85084.

22. Kern A, Khoury B, Frederickson A, Langevin R. The associations between childhood maltreatment and pregnancy complications: a systematic review and meta-analysis. J Psychosom Res. 2022;160:110985.

23. Hill A, Pallitto C, McCleary-Sills J, Garcia-Moreno C. A systematic review and meta-analysis of intimate partner violence during pregnancy and selected birth outcomes. Int J Gynecol Obstet. 2016;133(3):269–76.

24. Alvarez-Segura M, Garcia-Esteve L, Torres A, Plaza A, Imaz ML, Hermida-Barros L, et al. Are women with a history of abuse more vulnerable to perinatal depressive symptoms? A systematic review. Arch Womens Ment Health. 2014;17(5):343–57.

25. Howard LM, Oram S, Galley H, Trevillion K, Feder G. Domestic violence and perinatal mental disorders: a systematic review and meta-analysis. PLoS Med. 2013;10(5):e1001452.

26. Palo SK, Dubey S, Negi S, Sahay MR, Patel K, Swain S, et al. Effective interventions to ensure MCH (Maternal and Child Health) services during pandemic related health emergencies (Zika, Ebola, and COVID-19): A systematic review. PLoS One. 2022;17(5):e0268106.

27. Harville E, Xiong X, Buekens P. Disasters and perinatal health: a systematic review. Obstet Gynecol Surv. 2010;65(11):713–28.

28. Sanguanklin N, McFarlin BL, Park CG, et al. Effects of the 2011 flood in Thailand on birth outcomes and perceived social support. J Obstet Gynecol Neonatal Nurs. 2014;43(4):435–44.

29. Amjad S, Chojecki D, Osornio-Vargas A, Ospina MB. Wildfire exposure during pregnancy and the risk of adverse birth outcomes: a systematic review. Environ Int. 2021;156:106644.

30. Jayachandran S. Air quality and early-life mortality evidence from Indonesia's wildfires. J Hum Resour. 2009;44(4):916–54.

31. Ren JH, Chiang CLV, Jiang XL, Luo BR, Liu XH, Pang MC. Mental Disorders of pregnant and postpartum women after earthquakes: a systematic review. Disaster Med Public Health Prep. 2014;8(4):315–25.

32. Khatri GK, Tran TD, Fisher J. Prevalence and determinants of symptoms of antenatal common mental disorders among women who had recently experienced an earthquake: a systematic review. BMC Psychiatry. 2019;19(1):47.

33. Davies C, Segre G, Estradé A, Radua J, De Micheli A, Provenzani U, et al. Prenatal and perinatal risk and protective factors for psychosis: a systematic review and meta-analysis. Lancet Psychiatry. 2020 May;7(5):399–410.

34. Alibhai KM, Ziegler BR, Meddings L, Batung E, Luginaah I. Factors impacting antenatal care utilization: a systematic review of 37 fragile and conflict-affected situations. Confl Health. 2022;16(1):33.

35. Gopalan SS, Das A, Howard N. Maternal and neonatal service usage and determinants in fragile and conflict-affected situations: a systematic review of Asia and the Middle-East. BMC Wom Health. 2017;17:20.

36. Keasley J, Blickwedel J, Quenby S. Adverse effects of exposure to armed conflict on pregnancy: a systematic review. BMJ Glob Health. 2017;2(4):e000377.

37. Surdyk S, Itani M, Al-Lobaidy M, Kahale LA, Farha A, Dewachi O, et al. Weaponised uranium and adverse health outcomes in iraq: A systematic review. BMJ Glob Health. 2021;6(2):e004166.

38. Ladhani NNN, Shah PS, Murphy KE. Prenatal amphetamine exposure and birth outcomes: a systematic review and metaanalysis. Am J Obstet Gynecol. 2011;205(3):219.e1-219.e7.

39. Chomchai C, Na Manorom N, Watanarungsan P, Yossuck P, Chomchai S. Methamphetamine abuse during pregnancy and its health impact on neonates born at Siriraj Hospital, Bangkok, Thailand. Southeast Asian J Trop Med Public Health. 2004;35(1):228–31.

40. Conner SN, Bedell V, Lipsey K, Macones GA, Cahill AG, Tuuli MG. Maternal Marijuana Use and Adverse Neonatal Outcomes: A Systematic Review and Meta-analysis. Obstet Gynecol. 2016;128(4):713–23.

41. Hayes JS, Dreher MC, Nugent JK. Newborn outcomes with maternal marihuana use in Jamaican women. Pediatr Nurs. 1988;14(2):107–10.

42. Marchand G, Masoud AT, Govindan M, Ware K, King A, Ruther S, et al. Birth Outcomes of neonates exposed to marijuana in utero: a systematic review and meta-analysis. JAMA Netw Open. 2022;5(1):e2145653.

43. Pereira PP da S, Mata FAFD, Figueiredo ACMG, Silva RB, Pereira MG. maternal exposure to alcohol and low birthweight: a systematic review and meta-analysis. Rev Bras Ginecol Obstet. 2019;41:333–47.

44. Silva I da, Quevedo L de A, Silva RA da, Oliveira SS de, Pinheiro RT. Association between alcohol abuse during pregnancy and birth weight. Rev Saude Publica. 2011;45(5):864–9.

45. Jackson DJ, Batiste E, Rendall-Mkosi K. Effect of smoking and alcohol use during pregnancy on the occurrence of low birthweight in a farming region in South Africa. Paediatr Perinat Epidemiol. 2007;21(5):432–40.

46. Steane SE, Young SL, Clifton VL, Gallo LA, Akison LK, Moritz KM. Prenatal alcohol consumption and placental outcomes: a systematic review and meta-analysis of clinical studies. Am J Obstet Gynecol. 2021;225(6):607.e1-607.e22.

47. Pineles BL, Park E, Samet JM. Systematic review and meta-analysis of miscarriage and maternal exposure to tobacco smoke during pregnancy. Am J Epidemiol. 2014;179(7):807–23.

48. Marufu TC, Ahankari A, Coleman T, Lewis S. Maternal smoking and the risk of stillbirth: systematic review and meta-analysis. BMC Public Health. 2015;15(1):239.

49. Pineles BL, Hsu S, Park E, Samet JM. Systematic review and meta-analyses of perinatal death and maternal exposure to tobacco smoke during pregnancy. Am J Epidemiol. 2016;184(2):87–97.

50. Dadi AF, Miller ER, Mwanri L. Antenatal depression and its association with adverse birth outcomes in low and middle-income countries: A systematic review and meta-analysis. PLoS One. 2020;15(1):e0227323.

51. Dadi AF, Miller ER, Bisetegn TA, Mwanri L. Global burden of antenatal depression and its association with adverse birth outcomes: an umbrella review. BMC Public Health. 2020;20(1):173.

52. Karaçam Z, Kizilca Çakaloz D, Demir R. The impact of adolescent pregnancy on maternal and infant health in Turkey: Systematic review and meta-analysis. J Gynecol Obstet Hum Reprod. 2021;50(4):102093.

53. Grønvik T, Fossgard Sandøy I. Complications associated with adolescent childbearing in Sub-Saharan Africa: A systematic literature review and meta-analysis. PLoS ONE. 2018;13(9):e0204327.

54. Azevedo WF de, Diniz MB, Fonseca ESVB da, Azevedo LMR de, Evangelista CB. Complications in adolescent pregnancy: systematic review of the literature. Einstein São Paulo. 2015;13(4):618–26.

55. Williamson N. Motherhood in childhood: facing the adolescent pregnancy. New York: UNFPA; 2013.

56. Oosterwijk VNL, Molenaar JM, van Bilsen LA, Kiefte-de Jong JC. Ramadan fasting during pregnancy and health outcomes in offspring: a systematic review. Nutrients. 2021;13(10):3450.

57. Glazier JD, Hayes DJL, Hussain S, D'Souza SW, Whitcombe J, Heazell AEP, et al. The effect of Ramadan fasting during pregnancy on perinatal outcomes: a systematic review and meta-analysis. BMC Pregnancy Childbirth. 2018;18(1):421.

58. Berg RC, Underland V, Odgaard-Jensen J, Fretheim A, Vist GE. Effects of female genital cutting on physical health outcomes: a systematic review and meta-analysis. BMJ Open. 2014;4(11):e006316.

59. Lurie JM, Weidman A, Huynh S, Delgado D, Easthausen I, Kaur G. Painful gynecologic and obstetric complications of female genital mutilation/cutting: A systematic review and meta-analysis. PLoS Med. 2020;17(3):e1003088.

60. Obermeyer CM. The consequences of female circumcision for health and sexuality: an update on the evidence. Cult Health Sex. 2005 Sep 1;7(5):443–61.

61. Hirose A, Owolabi O, Imamura M, Okonofua F, Hussein J. Systematic review of obstetric care from a women-centered perspective in Nigeria since 2000. Int J Gynecol Obstet. 2017;136(1):13–8.

62. Banke-Thomas OE, Banke-Thomas AO, Ameh CA. Factors influencing utilisation of maternal health services by adolescent mothers in Low-and middle-income countries: a systematic review. BMC Pregnancy Childbirth. 2017;17(1):65.

63. Tesfaye G, Loxton D, Chojenta C, Semahegn A, Smith R. Delayed initiation of antenatal care and associated factors in Ethiopia: a systematic review and meta-analysis. Reprod Health. 2017;14(1):150.

64. Kassa GM, Arowojolu AO, Odukogbe AA, Yalew AW. Prevalence and determinants of adolescent pregnancy in Africa: a systematic review and Meta-analysis. Reprod Health. 2018;15(1):195.

65. Alene M, Yismaw L, Berelie Y, Kassie B, Yeshambel R, Assemie MA. Prevalence and determinants of unintended pregnancy in Ethiopia: a systematic review and meta-analysis of observational studies. PLoS One. 2020;15(4):e0231012.

66. Bain LE, Zweekhorst MBM, Buning T de C. Prevalence and determinants of unintended pregnancy in sub –Saharan Africa: a systematic review. Afr J Reprod Health. 2020;24(2):187–205.

67. Ali S, Khan U, Feroz A. Prevalence and determinants of anemia among women of reproductive age in developing countries. J Coll Physicians Surg Pak. 2020;30:177–86.

68. Ramaiya A, Kiss L, Baraitser P, Mbaruku G, Hildon Z. A systematic review of risk factors for neonatal mortality in adolescent mothers in sub-Saharan Africa. BMC Res Notes. 2014;7(1):750.

69. Ketema DB, Leshargie CT, Kibret GD, Assemie MA, Petrucka P, Alebel A. Effects of maternal education on birth preparedness and complication readiness among Ethiopian pregnant women: a systematic review and meta-analysis. BMC Pregnancy Childbirth. 2020;20(1):149.

70. Zhao J, Zhao Y, Du M, Binns CW, Lee AH. Maternal education and breastfeeding practices in China: A systematic review and meta-analysis. Midwifery. 2017;50:62–71.

71. Luo ZC, An N, Xu HR, Larante A, Audibert F, Fraser WD. The effects and mechanisms of primiparity on the risk of pre-eclampsia: a systematic review. Paediatr Perinat Epidemiol. 2007;21(s1):36–45.

72. Lee KW, Ching SM, Ramachandran V, Yee A, Hoo FK, Chia YC, et al. Prevalence and risk factors of gestational diabetes mellitus in Asia: a systematic review and meta-analysis. BMC Pregnancy Childbirth. 2018;18(1):494.

73. Nigussie J, Girma B, Molla A, Tamir T, Tilahun R. Magnitude of postpartum hemorrhage and its associated factors in Ethiopia: a systematic review and meta-analysis. Reprod Health. 2022;19(1):63.

74. Habitamu D, Goshu YA, Zeleke LB. The magnitude and associated factors of postpartum hemorrhage among mothers who delivered at Debre Tabor general hospital 2018. BMC Res Notes. 2019;12(1):618.

75. Temesgen MA. Magnitude of postpartum hemorrhage among women delivered at Dessie referral hospital, South Woll, Amhara region, Ethiopia. J Women's Health Care. 2017; 6(391):2167-0420.

76. Yang K, Wu J, Chen X. Risk factors of perinatal depression in women: a systematic review and meta-analysis. BMC Psychiatry. 2022;22(1):63.

77. Desta M, Amha H, Anteneh Bishaw K, Adane F, Assemie MA, Kibret GD, et al. Prevalence and predictors of uterine rupture among Ethiopian women: A systematic review and meta-analysis. PLoS One. 2020;15(11):e0240675.

78. Almeida LM, Caldas J, Ayres-de-Campos D, Salcedo-Barrientos D, Dias S. Maternal healthcare in migrants: a systematic review. Matern Child Health J. 2013;17(8):1346–54.

79. Gagnon AJ, Zimbeck M, Zeitlin J. Migration to western industrialised countries and perinatal health: A systematic review. Soc Sci Med. 2009;69(6):934–46.

80. Gagnon AJ, McDermott S, Rigol-Chachamovich J, et al. International migration and gestational diabetes mellitus: a systematic review of the literature and meta-analysis: Migration and gestational diabetes mellitus. Paediatr Perinat Epidemiol. 2011;25(6):575–92.

81. Harakow H, Hvidman L, Wejse C, Eiset AH. Pregnancy complications among refugee women: a systematic review. Acta Obstet Gynecol Scand. 2021;100(4):649–57.

82. Heslehurst N, Brown H, Pemu A, Coleman H, Rankin J. Perinatal health outcomes and care among asylum seekers and refugees: a systematic review of systematic reviews. BMC Med. 2018;16(1):89.

83. Gissler M, Alexander S, MacFarlane A, et al. Stillbirths and infant deaths among migrants in industrialized countries. Acta Obstet Gynecol Scand. 2009;88(2):134–48.

84. Fan S, Koski A. The health consequences of child marriage: a systematic review of the evidence. BMC Public Health. 2022;22(1):309.

85. Haider BA, Olofin I, Wang M, Spiegelman D, Ezzati M, Fawzi WW. Anaemia, prenatal iron use, and risk of adverse pregnancy outcomes: systematic review and meta-analysis. BMJ. 2013;346: f3443.

86. Mahomed K. Folate supplementation in pregnancy. Cochrane Database Syst Rev. 1997;CD000183.

87. Mahomed K. Iron supplementation in pregnancy. Cochrane Database Syst Rev. 2000; CD000117.

88. Shi Q, Leng W, Wazir R, et al. Intravenous iron sucrose versus oral iron in the treatment of pregnancy with iron deficiency anaemia: a systematic review. Gynecol Obstet Invest. 2015;80(3):170–8.

89. Dorsamy V, Bagwandeen C, Moodley J. The prevalence, risk factors and outcomes of anaemia in South African pregnant women: a systematic review and meta-analysis. Syst Rev. 2022;11(1):16.

90. Peña-Rosas JP, De-Regil LM, Dowswell T, Viteri FE. Daily oral iron supplementation during pregnancy. Cochrane Database Syst Rev. 2012;12:CD004736.

91. Lewkowitz AK, Gupta A, Simon L, Sabol BA, Stoll C, Cooke E, et al. Intravenous compared with oral iron for the treatment of iron-deficiency anemia in pregnancy: a systematic review and meta-analysis. J Perinatol. 2019;39(4):519–32.

92. Peña-Rosas JP, De-Regil LM, Garcia-Casal MN, Dowswell T. Daily oral iron supplementation during pregnancy. Cochrane Database Syst Rev. 2015; CD004736.

93. Jahanfar S, Howard LM, Medley N. Interventions for preventing or reducing domestic violence against pregnant women. Cochrane Database Syst Rev. 2014; CD009414.

94. Chamberlain C, O'Mara-Eves A, Porter J, et al. Psychosocial interventions for supporting women to stop smoking in pregnancy. Cochrane Database Syst Rev. 2017; CD001055.

95. Chowdhary N, Sikander S, Atif N, et al. The content and delivery of psychological interventions for perinatal depression by non-specialist health workers in low and middle income countries: a systematic review. Best Pract Res Clin Obstet Gynaecol. 2014;28(1):113–33.

96. Leng LL, Yin XC, Ng SM. Mindfulness-based intervention for clinical and subthreshold perinatal depression and anxiety: a systematic review and meta-analysis of randomized controlled trial. Compr Psychiatry. 2023;122:152375.

97. Munodawafa M, Mall S, Lund C, Schneider M. Process evaluations of task sharing interventions for perinatal depression in low and middle income countries (LMIC): a systematic review and qualitative meta-synthesis. BMC Health Serv Res. 2018;18(1):205.

98. Dixon S, Dantas JAR. Best practice for community-based management of postnatal depression in developing countries: a systematic review. Health Care Women Int. 2017;38(2):118–43.

99. Yin J, Nisar A, Waqas A, Guo Y, Qi WL, Wang D, et al. Psychosocial interventions on perinatal depression in China: A systematic review and meta-analysis. J Affect Disord. 2020;271:310–27.

100. Jidong DE, Husain N, Roche A, Lourie G, Ike TJ, Murshed M, et al. Psychological interventions for maternal depression among women of African and Caribbean origin: a systematic review. BMC Wom Health. 2021;21(1):83.

101. Huang R, Yan C, Tian Y, Lei B, Yang D, Liu D, et al. Effectiveness of peer support intervention on perinatal depression: a systematic review and meta-analysis. J Affect Disord. 2020;276:788–96.

102. Shortis E, Warrington D, Whittaker P. The efficacy of cognitive behavioral therapy for the treatment of antenatal depression: a systematic review. J Affect Disord. 2020;272:485–95.

103. Nigusie A, Azale T, Yitayal M. Institutional delivery service utilization and associated factors in Ethiopia: a systematic review and meta-analysis. BMC Pregnancy Childbirth. 2020;20(1):364.

104. Simkhada B, Teijlingen ER van, Porter M, Simkhada P. Factors affecting the utilization of antenatal care in developing countries: systematic review of the literature: Factors affecting the utilization of antenatal care. J Adv Nurs. 2008;61(3):244–60.

105. Moyer CA, Mustafa A. Drivers and deterrents of facility delivery in sub-Saharan Africa: a systematic review. Reprod Health. 2013;10(1):40.

106. Tekelab T, Chojenta C, Smith R, Loxton D. Factors affecting utilization of antenatal care in Ethiopia: A systematic review and meta-analysis. PLoS One. 2019;14(4):e0214848.

107. Lassi ZS, Kedzior SG, Bhutta ZA. Community-based maternal and newborn educational care packages for improving neonatal health and survival in low- and middle-income countries. Cochrane Database Syst Rev. 2019; CD007647.

108. Habtewold TD, Mohammed SH, Endalamaw A, et al. Higher educational and economic status are key factors for the timely initiation of breastfeeding in Ethiopia: a review and meta-analysis. Acta Paediatr. 2020;109(11):2208–18.

109. Oringanje C, Meremikwu MM, Eko H, Esu E, Meremikwu A, Ehiri JE. Interventions for preventing unintended pregnancies among adolescents. Cochrane Database Syst Rev. 2016; CD005215.

110. Mbuagbaw L, Medley N, Darzi AJ, Richardson M, Garga KH, Ongolo-Zogo P. Health system and community level interventions for improving antenatal care coverage and health outcomes. Cochrane Database Syst Rev. 2015; CD010994.

111. Jacobs W, Downey LE. Impact of conditional cash transfer programmes on antenatal care service uptake in low and middle-income countries: a systematic review. BMJ Open. 2022;12(11):e064673.

112. Vanhuyse F, Stirrup O, Odhiambo A, Palmer T, Dickin S, Skordis J, et al. Effectiveness of conditional cash transfers (Afya credits incentive) to retain women in the continuum of care during pregnancy, birth and the postnatal period in Kenya: a cluster-randomised trial. BMJ Open. 2022;12(1):e055921.
